# Allele-specific binding of RNA-binding proteins reveals functional genetic variants in the RNA

**DOI:** 10.1038/s41467-019-09292-w

**Published:** 2019-03-22

**Authors:** Ei-Wen Yang, Jae Hoon Bahn, Esther Yun-Hua Hsiao, Boon Xin Tan, Yiwei Sun, Ting Fu, Bo Zhou, Eric L. Van Nostrand, Gabriel A. Pratt, Peter Freese, Xintao Wei, Giovanni Quinones-Valdez, Alexander E. Urban, Brenton R. Graveley, Christopher B. Burge, Gene W. Yeo, Xinshu Xiao

**Affiliations:** 1 Department of Integrative Biology and Physiology, UCLA, Los Angeles, CA 90095 USA; 2 Department of Bioengineering, UCLA, Los Angeles, CA 90095 USA; 3 Molecular, Cellular and Integrative Physiology Interdepartmental Program, UCLA, Los Angeles, CA 90095 USA; 4 Department of Psychiatry and Behavioral Sciences, Department of Genetics, Stanford University School of Medicine, Palo Alto, CA 94305 USA; 5 Department of Cellular and Molecular Medicine, UCSD, La Jolla, CA 92093 USA; 6 Institute for Genomic Medicine, UCSD, La Jolla, CA 92093 USA; 7 Department of Biology, MIT, Cambridge, MA 02139 USA; 8 Department of Genetics and Genome Sciences, Institute for Systems Genomics, UConn Health, Farmington, CT 06030 USA; 9 Department of Physiology, Yong Loo Lin School of Medicine, National University of Singapore, Singapore, 117593 Singapore; 10 Molecular Engineering Laboratory, A*STAR, Singapore, 138673 Singapore; 11 Molecular Biology Institute, UCLA, Los Angeles, CA 90095 USA

## Abstract

Allele-specific protein-RNA binding is an essential aspect that may reveal functional genetic variants (GVs) mediating post-transcriptional regulation. Recently, genome-wide detection of in vivo binding of RNA-binding proteins is greatly facilitated by the enhanced crosslinking and immunoprecipitation (eCLIP) method. We developed a new computational approach, called BEAPR, to identify allele-specific binding (ASB) events in eCLIP-Seq data. BEAPR takes into account crosslinking-induced sequence propensity and variations between replicated experiments. Using simulated and actual data, we show that BEAPR largely outperforms often-used count analysis methods. Importantly, BEAPR overcomes the inherent overdispersion problem of these methods. Complemented by experimental validations, we demonstrate that the application of BEAPR to ENCODE eCLIP-Seq data of 154 proteins helps to predict functional GVs that alter splicing or mRNA abundance. Moreover, many GVs with ASB patterns have known disease relevance. Overall, BEAPR is an effective method that helps to address the outstanding challenge of functional interpretation of GVs.

## Abstract (web summary)

Differential binding of RNA-binding proteins mediated by genetic variants (GVs) can influence posttranscriptional regulation. Here, the authors develop BEAPR, a computational approach to identify allele-specific binding events in eCLIP-Seq data.

## Introduction

Facilitated by recent technological advances, numerous human genomes are being sequenced, cataloging an unprecedented amount of genetic variants (GVs)^1^. A major challenge exists in identifying and interpreting potentially functional GVs. Disease-associated GVs often reside in non-coding regions, such as introns or 3′-untranslated regions (UTRs), making it especially challenging for functional interpretation^2^. It is increasingly appreciated that many GVs in the introns or 3′- UTRs may affect RNA processing or messenger RNA (mRNA) turnover^3^. Thus, methodologies that can effectively capture functional GVs in these post-transcriptional processes are in great demand.

RNA-binding proteins (RBPs) are core players in post-transcriptional gene regulation^4,5^. A large number of RBPs exert their function via sequence-specific protein–RNA interaction. The sequence specificity of RBPs implies that GVs may disrupt RBP recognition of RNA substrates. Specifically, the alternative alleles of a GV may confer different binding specificity for an RBP, thus causing allele-specific functional consequences^6^.

To detect ASB of a specific RBP, one powerful method is to examine global binding sites of the RBP. If a heterozygous GV is present within the binding site, allelic bias of the GV in the protein-bound RNA directly suggests existence of ASB. The advantage of this analysis is that the alternative alleles of a GV are examined in the same cellular environment in the same subject. Thus, the method controls for tissue conditions, trans-acting factors, global epigenetic effects, and other environmental influences.

To carry out genome-wide ASB analyses, it is necessary to capture global protein–RNA interaction in a sequence-specific manner. Ultraviolet crosslinking and immunoprecipitation followed by sequencing (CLIP-Seq) is a most-often used method for this type of global profiling^7^. Recently, the enhanced CLIP (eCLIP) protocol was developed that significantly improves the efficiency and sensitivity of CLIP^8^. As a result of the improved efficiency, multiple biological replicates of eCLIP can be generated for the same experiment. In addition, each eCLIP assay is accompanied by a size-matched input (SMInput) sample as a stringent control for non-specific binding. The ENCODE project generated hundreds of eCLIP-Seq data sets for 154 RBPs in two cell lines, HepG2 and K562^9^. These data sets afford an invaluable opportunity to examine ASB patterns and shed light on the functions of GVs in post-transcriptional regulation.

However, ASB analysis is challenging in that it entails accurate quantification of single nucleotides in sequencing reads, which is easily confounded by possible inherent biases in the CLIP protocol and the limited sequencing depth available for most CLIP data sets. Nevertheless, the unique advantages of eCLIP-Seq, such as the availability of biological replicates and SMInput samples, offer an opportunity to accurately identify ASB events. Thus far, no computational method is available that leverages these unique features of eCLIP for ASB detection. Here, we present a new method called BEAPR (Binding Estimation of Allele-specific Protein–RNA interaction) for this purpose. BEAPR controls for inherent bias in crosslinking using the SMInput samples, and tests for significant binding bias by taking into account the variability in the data as manifested in the biological replicates. We show that BEAPR outperforms standard methods for allele-specific analyses of read counts. Importantly, BEAPR is robust to overdispersion in the sequencing data and its performance is consistent across different ranges of read coverage. Applied to the ENCODE eCLIP-Seq data sets, BEAPR identifies thousands of ASB events. Supported by experimental validations, these ASB events include many that can potentially cause splicing changes, alter mRNA abundance, or explain the functional consequences of disease-associated GVs. Together, our results suggest that BEAPR is an effective method for ASB detection and can serve as a fundamental tool to predict functional GVs in post-transcriptional gene regulation.

## Results

### Identification of allele-specific binding of RBPs by BEAPR

**Fig. 1:**
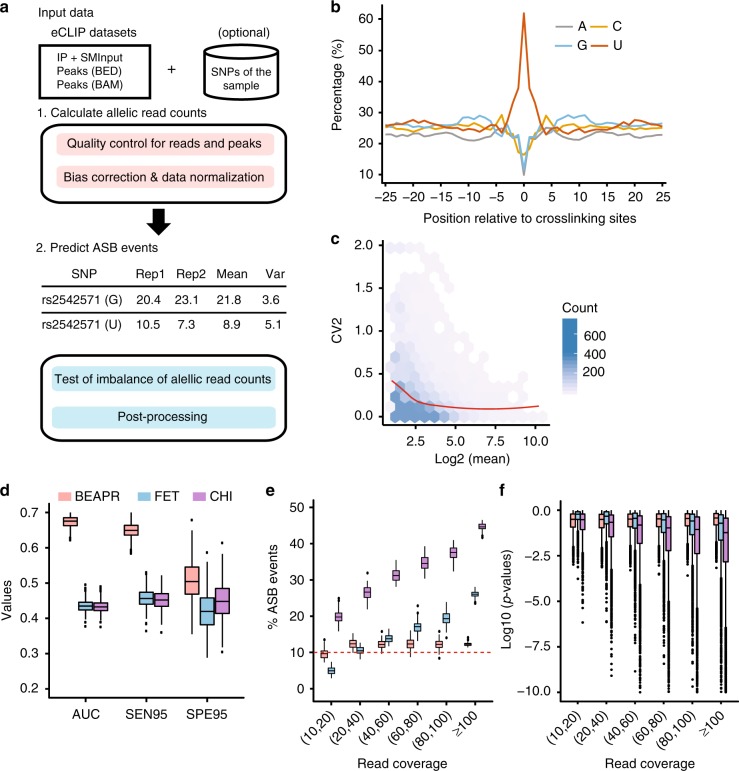
Overview of BEAPR (Binding Estimation of Allele-specific Protein–RNA interaction) and its performance. **a** Overall work flow of BEAPR. See Methods for details. **b** Crosslinking-induced bias in the SMInput sample of RBFOX2 (HepG2 cells). *Y*-axis shows the relative % of each nucleotide observed at each position relative to the crosslinking site (*x* = 0). **c** The square of the coefficient of variation (CV2) plotted as a function of the observed allelic read counts (mean of the two replicates) in the RBFOX2 enhanced crosslinking and immunoprecipitation (eCLIP) data in HepG2 cells (see Methods). **d** Performance comparison of three methods using simulated data (with simulated crosslinking-induced biases) and true allelic ratio of 0.8 for allele-specific binding (ASB). Data derived from 1000 simulation experiments each encompassing 5000 single-nucleotide variants (SNVs) (10% of which being ASB). FET: Fisher’s exact test; CHI: *χ*^2^ test; AUC: area under the curve of the precision-recall curve. SEN95: sensitivity at 95% specificity; SPE95: specificity at 95% sensitivity. **e** Percentage of ASB events among all tested SNVs by the three methods using simulated data as in **d**. The *x*-axis shows different read coverage bins (using average read coverage of each SNV in two simulated replicates). The red dashed line corresponds to the 10% value, that is, the percentage of true ASB events in the simulation. **f** Box plots of *p* values calculated by the three methods at different levels of read coverage. Boxplot center lines indicate the median and the boxes extend to lower and upper quartiles with whiskers depicting 1.5 interquartile range (IQR). The discrete points are the outliers. (Source data are provided as a Source Data file)

BEAPR analyzes eCLIP-Seq data to identify ASB events in protein–RNA interaction. The standard eCLIP-Seq protocol generates an input control sample (SMInput) and two biological replicates of eCLIP samples^9^. As illustrated in Fig. 1a (see Methods for details), BEAPR takes as input mapped reads and peak calls from these data sets. It first identifies heterozygous single-nucleotide variants (SNVs) that show bi-allelic expression in the SMInput reads. An optional input is a list of sample-specific heterozygous SNVs. If provided, this list will be combined with BEAPR-identified heterozygous SNVs. Although genome-sequencing or genotyping data may exist for the specific samples, identification of SNVs using SMInput reads may complement these data (see below).

A unique feature of BEAPR is the estimation of crosslinking-induced sequence bias, using the SMInput data of each eCLIP experiment. As an example, Fig. 1b shows the bias estimation in the RBFOX2 data generated from HepG2 cells. The enrichment of uracils at the crosslinking sites is consistent as that reported by previous CLIP studies^10^. This bias, specifically estimated for each eCLIP experiment, is used to normalize the allele-specific read counts of each SNV. Subsequently, BEAPR employs an empirical Gaussian distribution to model the normalized read counts, with the expected variance estimated using a regression model (Fig. 1c, see Methods). BEAPR tests whether the normalized read counts of the alternative alleles of an SNV are significantly different (i.e., existence of ASB). The predicted ASB events were subject to several filters to remove those located in homopolymeric or repetitive regions, regions with inherent mapping bias, or event in genes with allele-specific gene expression identified using RNA-Seq data of the same cell type (if exist) (see Methods).

### Evaluation of BEAPR performance using simulated data

We first generated simulated data to evaluate the performance of BEAPR. Specifically, we carried out 1000 simulation experiments. In each experiment, 5000 heterozygous SNVs were included, each of which was assigned a total read coverage and read counts for two alternative alleles, with two simulated biological replicates. The total read coverage of the SNVs and the variance across replicates were sampled randomly from the actual read coverage distributions of SNVs of an ENCODE eCLIP data set (SRSF1 in K562 cells, Supplementary Figure 1). The read counts for alternative alleles of an SNV were determined using a zero-truncated negative binomial distribution given the simulated total read coverage and an expected allelic ratio *r* (read count of major allele/total read count). The value of *r* was set to be 0.5 for 90% of the simulated SNVs in each experiment. The other 10% were simulated ASB events (i.e., true positives) with an *r* value of 0.7, 0.8, or 0.9 in different experimental settings. To identify ASB events, a minimum total read coverage (per replicate) of 10 was required for all methods.

The performance of BEAPR was compared to those of two other methods: *χ*^2^ test and Fisher’s exact test, both were used to detect allelic imbalance in read count data^11,12^. Since these methods cannot model the variability between biological replicates, read counts from replicates were combined when using these methods. It should be noted that crosslinking-induced sequence bias was not taken into consideration by these methods. Thus, in the first simulation, no crosslinking-induced sequence bias was simulated. Performance was assessed by the precision-recall curves, sensitivity and specificity (see Methods). As shown in Supplementary Figure 2, BEAPR achieved the highest area under the curve (AUC) in the precision-recall curves, and the highest sensitivity and specificity among the three methods. In general, ASB identification is challenging if the true allelic ratio is close to 0.5, given limited read coverage (Supplementary Figure 1). The performance of the three methods deteriorates at smaller *r* values (e.g., 0.7). Next, we included crosslinking-induced bias in the simulations (see Methods). As expected, the performance of *χ*^2^ test and Fisher’s exact test declined. In contrast, BEAPR’s performance remained largely unchanged (Fig. 1d, Supplementary Figure 3). Overall, BEAPR outperformed the other methods consistently at all tested allelic ratios.

### BEAPR accounts for overdispersion in allelic read counts

Overdispersion exists if the variance of the count data is underestimated, which may lead to enrichment of very small *p* values and false-positive predictions. In the simulation study, we examined whether the results of different methods reflected overdispersion in the data. Considering the fact that other methods cannot handle crosslinking-induced bias, we used simulations without such bias. Figure 1e and Supplementary Figure 2 show that the percentage of predicted ASB events (false discovery rate (FDR) <10%) among all tested SNVs increased substantially at higher read coverages based on *χ*^2^ test and Fisher’s exact test. This overdispersion is largely due to inflated *p* values calculated by these methods at higher read coverages (Fig. 1f, Supplementary Figure 2c, f, i). In contrast, BEAPR demonstrated a relatively stable percentage of ASB and, consistently, stable *p* values across different ranges of read coverage. Next, we examined the performance of the three methods in handling SNVs with different levels of read count variance (see Methods). Again, BEAPR outperformed the other methods consistently and its performance is robust given high variance (compared to moderate variance level, Supplementary Figure 4). In contrast, Fisher’s exact test and *χ*^2^ test performed poorly for SNVs with high variances (Supplementary Figure 4). The results suggest that BEAPR is robust to the variance in the input read count data, which contributed to its superior performance.

### Analysis of ENCODE eCLIP-Seq data using BEAPR

We obtained eCLIP-Seq data of 154 RBPs derived from HepG2 or K562 cells as part of the ENCODE project^9^. Each RBP had two biological replicates of eCLIP and one SMInput control. The reads were pre-processed and mapped using STAR as described previously^8^. eCLIP peaks were identified using CLIPper^13^. In this work, eCLIP peaks were retained for subsequent analyses if the read coverage in at least one replicate is ≥4-fold of that in the corresponding region in the SMInput. This fold-change cutoff was chosen by comparing the ASB results using a range of cutoff values (Supplementary Figure 5).

**Fig. 2:**
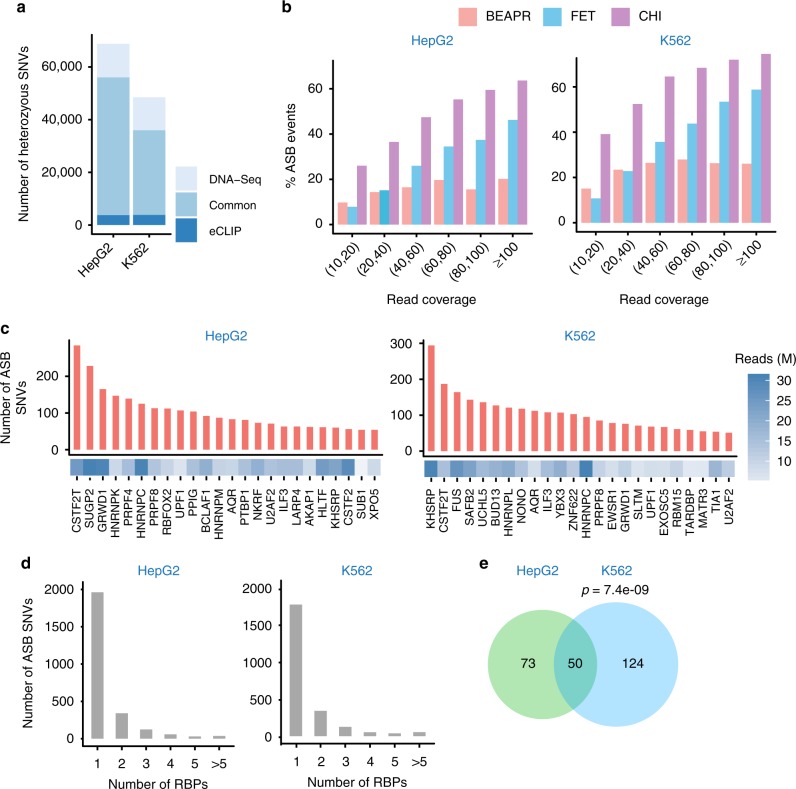
Allele-specific binding (ASB) events identified in the enhanced crosslinking and immunoprecipitation (eCLIP) data of HepG2 and K562 cells. **a** Number of heterozygous single-nucleotide variants (SNVs) identified via whole-genome DNA sequencing or eCLIP. Those that are common to both methods are illustrated. **b** Percentage of ASB events among all testable SNVs in each read coverage bin. The average read coverage of each SNV in the two replicated eCLIP experiments is shown here. A minimum read coverage of 10 was required. **c** Number of ASB events identified for each RNA-binding protein (RBP) in HepG2 and K562 cells. Only RBPs with ≥50 ASB events are shown. The number of usable eCLIP-Seq reads (in millions (M)) is shown for each RBP. **d** Number of ASB SNVs associated with one or more than one RBPs. **e** The overlap of ASB SNVs between HepG2 and K562. Only heterozygous SNVs common to the two cell lines are included. *P* value was calculated by Fisher’s exact test. (Source data are provided as a Source Data file)

Given the above-defined eCLIP peak regions, BEAPR proceeds to identify heterozygous SNVs in these regions, which will be combined with sample-specific SNVs if provided by the user. For the ENCODE data sets, we identified heterozygous SNVs in HepG2 and K562 cells using both the eCLIP data and whole-genome sequencing data^14,15^ (see Methods). Within the eCLIP peak regions, the heterozygous SNVs identified via the two methods overlapped substantially (Fig. 2a, Supplementary Figure 5). Around 93.1% of eCLIP-derived SNVs in HepG2 (89.2% in K562) were also identified in the respective whole-genome sequencing data. About 80.4% of SNVs located in eCLIP peaks and predicted by whole-genome sequencing of HepG2 (71.9% for K562) were also identified by our method. Thus, if assuming whole-genome sequencing as the ground truth, eCLIP-based SNV identification achieved a precision of 93.1 and 89.2%, and a sensitivity of 80.4 and 71.9%, in HepG2 and K562 cells for SNVs located in eCLIP peaks, respectively. Furthermore, we experimentally confirmed five heterozygous SNVs that were identified in the eCLIP data, but missed by whole-genome sequencing (Supplementary Figure 6, Supplementary Data 1), supporting the validity of these SNVs. Our results suggest that BEAPR can be used to identify heterozygous SNVs using eCLIP data alone, if genotyping or genome sequencing data are not available for the specific sample.

Next, we asked whether the prediction of ASB by BEAPR reflected overdispersion in the ENCODE eCLIP data. To this end, we examined the percentage of ASB events (%ASB) among all testable SNVs, with ASB events called by requiring FDR <10%. This percentage is expected to be independent of read coverage in the absence of overdispersion. As shown in Fig. 2b, BEAPR yielded relatively stable %ASB at different levels of read coverage. Similar to the observations in simulated data, *χ*^2^ test and Fisher’s exact test suffered from overdispersion, as manifested by the higher %ASB at higher levels of read coverage. The results suggest that these methods underestimate the variance of allelic read counts in the eCLIP data and tend to produce false-positive predictions given high read coverage.

### ASB events identified in ENCODE eCLIP-Seq data

For all RBPs with eCLIP data, a total of 3706 and 3783 ASB events were identified in the HepG2 and K562 cells, respectively. All events passed the posterior filers that remove potential artifacts due to repetitive regions, mapping bias, or allele-specific gene expression (Supplementary Figure 7a. see Methods). The RBPs with more than 50 predicted ASB events are illustrated in Fig. 2c and the results for all RBPs are shown in Supplementary Figure 7 and Supplementary Data 2. The number of ASB events associated with different RBPs varied greatly. This variation may be accounted for by multiple factors, such as sequencing depth, number of eCLIP peaks, and the binding specificity of the RBP. The genomic distribution of ASB events often reflects known functions of the RBPs (Supplementary Figure 8a). For example, proteins known to regulate RNA stability, such as UPF1^16^, showed ASB enrichment in the 3′-UTR regions. ASB events of known splicing regulators^17^, such as RBFOX2, PTBP1, PRPF8, U2AF1, and heterogeneous nuclear ribonucleoproteins (hnRNPs), were enriched in the intronic regions.

In general, consistency was observed between the genomic distributions of ASB events and eCLIP peaks (Supplementary Figure 8b). Nonetheless, the existence of an ASB event depends on two factors: existence of an eCLIP peak and a heterozygous SNV in the peak. As a result, regions (such as coding exons) with a paucity of SNVs (Supplementary Figure 8c) had relatively less ASB events compared to eCLIP peaks (Supplementary Figure 8b). In contrast, introns were associated with relatively more ASB events than eCLIP peaks for some RBPs (Supplementary Figure 8b). Nonetheless, the occurrence frequency of ASB among all testable SNVs in each region is relatively constant (Supplementary Figure 8d), suggesting a similar enrichment of ASB events across regions.

The above ASB events occurred in 2552 and 2385 SNVs in HepG2 and K562, respectively. As shown in Fig. 2d, 76.9 and 77.6% of these SNVs were associated with ASB of one RBP in HepG2 and K562, respectively, suggesting that ASB events are mostly RBP-specific. Nevertheless, multiple RBPs may interact with each other leading to common eCLIP peaks and ASB events. In addition, RBPs with similar binding motifs may share eCLIP peaks and ASB events, the examples of which are shown in Supplementary Figure 9.

Next, we compared the ASB events between the two cell lines. A total of 760 heterozygous SNVs were testable for ASB of common RBPs in both cell lines. Among these SNVs, 123 and 174 were identified with ASB patterns in HepG2 and K562, respectively, with 50 shared by the two cell lines (Fig. 2e, *p* = 7.4e − 09, Fisher’s exact test). Thus, these 50 SNVs could function in a cell-type-independent manner, at least between the two cell lines tested in this study.

### SNVs with ASB patterns disrupt RBP binding motifs

**Fig. 3:**
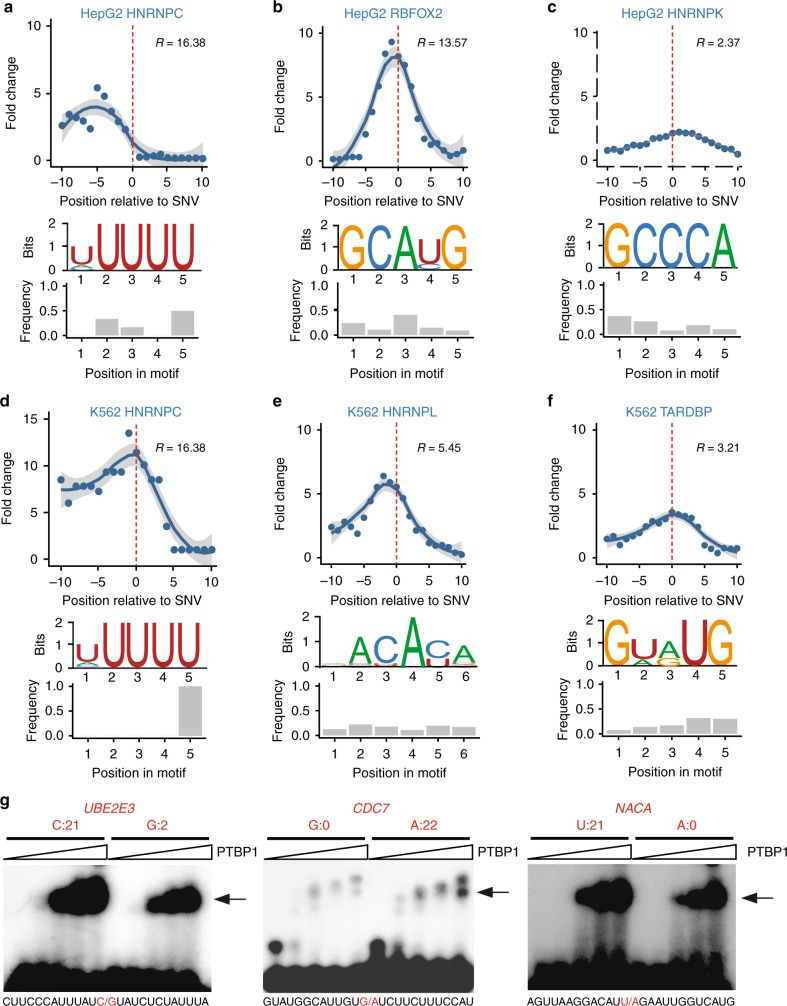
Bioinformatic and experimental validation of allele-specific binding (ASB). **a**–**f** Enrichment of RNA Bind-n-Seq (RBNS) motifs in the regions around ASB single-nucleotide variants (SNVs) (*x* = 0) of each RNA-binding protein (RBP) (upper panel). *Y*-axis shows fold change in the enrichment relative to randomly chosen control regions (Methods). Ten sets of controls were constructed, with the regression curve and 95% confidence interval of the average fold change shown in the panel. RBNS motifs used in the analysis are shown (middle panel). The relative frequency of the ASB SNVs overlapping each motif position is shown in the bar graph (lower panel). **g** Electrophoretic mobility shift assays (EMSA) results of PTBP1 binding to its ASB targets. Alternative alleles of the ASB SNVs were synthesized, as labeled above the gel images. Read count (sum of two replicates) for each allele in the enhanced crosslinking and immunoprecipitation (eCLIP) data of PTBP1 (HepG2 cells) is shown. (Images are cropped, with uncropped images in Supplementary Figure 16.) The sequences of the synthetic RNA fragments are shown below each gel image, where the ASB SNV is highlighted in red. The arrow indicates RNA–protein complex. Increasing concentrations of PTBP1 were used in different lanes of the gel image (from left to right: 0, 0.6, 1.2, 2.5, and 5 μg). (Source data are provided as a Source Data file)

For an RBP with specific sequence preference, it is expected that ASB patterns may arise if an SNV disrupts its binding motif. Thus, the localization of ASB SNVs near known RBP binding motifs serves as a strong indicator of the validity of the predicted ASB. We obtained binding motifs identified by the RNA Bind-n-Seq (RBNS) assay as part of the ENCODE project^9^. RBNS quantifies the binding specificity of an RBP to a k-mer sequence using the *R* value^18,19^. To focus on RBPs with relatively high binding specificity, we required an RBP to have at least one k-mer sequence with *R* ≥ 2. Among all RBPs with at least 50 ASB events and at least 30 events in annotated genes, five RBPs, hnRNPC, hnRNPK, hnRNPL, RBFOX2, and TARDBP, had RBNS k-mers that passed this requirement. For these RBPs, we analyzed the occurrence of the RBNS k-mers in the flanking regions of ASB SNVs. Compared to control regions (see Methods), the ASB flanking regions were enriched with RBNS motifs, and, importantly, the ASB loci were located in close proximity to the k-mer enrichment peaks (Fig. 3a–f). Interestingly, the fold enrichment of RBNS k-mers in ASB regions relative to control regions is often higher for proteins with higher *R* values. These results strongly support the validity of the predicted ASB events. Note that the enrichment of RBNS motifs was lower if no correction for crosslinking-induced bias was carried out (Supplementary Figure 10), supporting the importance of this step. We observed that the specific nucleotide position disrupted by the ASB SNVs varied for different RBPs (Fig. 3a–f), which may depend on the specific binding property of the RBPs and the specific allelic sequences of ASB SNVs.

For RBPs with ≥50 ASB events (and ≥30 in annotated genes) but without specific RBNS motifs, we identified the top five most-frequent pentamers in the 21-mer region centered at the ASB SNV of each RBP (Supplementary Figure 11). Most of these proteins do not have known motifs in the literature, likely due to low sequence specificity. However, for a small number of RBPs with known binding preference, such as PTBP1 (CU-rich motif^20^), the enriched pentamers are largely consistent with their known motifs. In addition, the positional distribution of these enriched pentamers often showed biases relative to the loci of ASB SNVs. It is expected that proteins with low binding specificity do not demonstrate strong signals in this analysis, which may explain the lack of positional bias for some RBPs. These results again support that ASB analysis can effectively capture specific RBP binding sites and allelic biases in protein–RNA interaction.

### Experimental validation of ASB events

To provide direct experimental support that ASB SNVs alter the binding of RBPs, we carried out electrophoretic mobility shift assays (EMSA, or gel shift) on randomly selected ASB events of PTBP1 (Fig. 3g, Supplementary Figure 12, Supplementary Data 1). This protein was chosen since it is relatively easy to purify. To confirm that the ASB SNVs alter the binding of PTBP1, two versions of each target RNA were synthesized harboring the alternative alleles of the SNV. As shown in Fig. 3g, the binding of PTBP1 to target RNAs was stronger with increasing protein input. The alternative alleles of the SNVs demonstrated visible differences in their binding to PTBP1. Specifically, the alleles with the stronger gel shift signals were consistent with the alleles that had more eCLIP reads, supporting the validity of the predicted ASB events.

Together, the above results support the validity of our ASB identification method. Since ASB serves as a direct indicator of functional SNVs, it is expected that ASB patterns can inform functional interpretations of GVs. Next, we examined whether the above ASB analysis captured functional SNVs in regulating alternative splicing and mRNA abundance.

### SNVs subject to ASB may cause splicing alteration

**Fig. 4:**
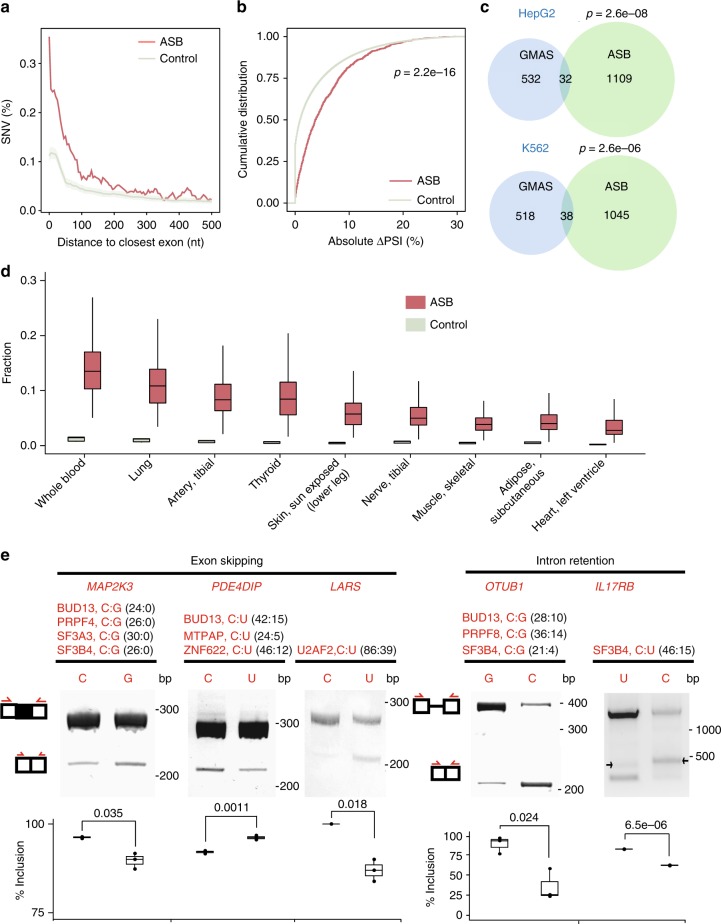
Functional relevance of allele-specific binding (ASB) single-nucleotide variants (SNVs) in splicing regulation. **a** Distance of intronic ASB SNVs to the nearest splice sites. Controls consist of randomly chosen SNVs in the same introns. A total of 100 sets of controls were constructed, with the average and standard deviation shown in the plot. **b** Absolute change in the percent spliced-in (PSI) values of exons associated with ASB events of splicing factors upon knockdown of the respective splicing factor in HepG2 or K562 cells. Controls were random intronic SNVs in the same introns. *P* value was calculated by the Kolmogorov–Smirnov test. **c** Overlap between ASB SNVs of splicing factors and heterozygous SNVs associated with genetically modulated alternative splicing (GMAS) events in the genes harboring ASB SNVs. *P* values were calculated by the hypergeometric test (see Methods). **d** Fraction of ASB SNVs located in GTEx splicing quantitative trait loci (sQTL) exons or within 500 nucleotide (nt) in their flanking introns among the union of ASB SNVs of all splicing factors in the HepG2 or K562 data. This fraction was calculated for each sample individually, with the distribution of all samples in a tissue shown in the box plots. Control: fraction of randomly chosen heterozygous SNVs within genes in the above regions in each sample. **e** Splicing reporter validation of the function of ASB events. Three exon skipping events and two intron retention events are included. The gene names with the ASB events, the associated RNA-binding proteins (RBPs), RNA alleles of the ASB SNV, and their read counts in enhanced crosslinking and immunoprecipitation (eCLIP) are shown. (Images are cropped, with uncropped images in Supplementary Figure 16.) The red arrows in the exon–intron diagrams indicate positions of PCR primers. Inclusion level (three biological replicates) of the exon or intron is shown below each gel image. *P* values were calculated by Student’s *t-*test. Note that the *IL17RB* minigene had alternative splice sites in the intron, which led to the extra bands (black arrows). Boxplot center lines indicate the median and the boxes extend to lower and upper quartiles with whiskers depicting 1.5 interquartile range (IQR). (Source data are provided as a Source Data file)

The functional consequence of the ASB event depends on the function of the RBP. Since many RBPs in this study are known splicing factors, we examined whether some ASB events may alter splicing. We collected all ASB events of known splicing factors in each cell line (37 in HepG2 and 31 in K562). First, we examined the distance of intronic SNVs with ASB by splicing factors to the nearest splice site. Compared to randomly selected SNVs in the same introns (see Methods), ASB SNVs were significantly closer to the splice sites (Fig. 4a). Next, to verify that the ASB events are associated with regulatory targets of the splicing factors, we analyzed splicing changes of the associated exons upon knockdown (KD) of the corresponding RBP using ENCODE RNA-Seq data in HepG2 or K562 cells^9^. Compared to random controls (see Methods), ASB-associated exons had a significantly larger change in the percent spliced-in (PSI) values upon KD of the splicing factors (Fig. 4b). This result supports the hypothesis that ASB-associated exons are bona fide targets of the splicing factors. It should be noted that PSI changes of the ASB target exons upon splicing factor KD are not expected to be very large in magnitude because the nature of ASB implicates that only one of two alleles of the endogenous SNV is bound strongly by the corresponding RBP.

### ASB SNVs overlap genetically modulated splicing events

If an ASB event is functional, we expect that the associated exon or gene is under *cis*-regulation by this SNV. For splicing factors, such ASB events will lead to allele-specific alternative splicing (i.e., genetically modulated alternative splicing, GMAS). Using our previous methods^6,21^, we identified GMAS events in RNA-Seq data of control HepG2 and K562 cells generated by the ENCODE project (see Methods). Using these data, we observed that SNVs with ASB patterns of splicing factors are significantly enriched in the GMAS exons or within 500 nt from their exon–intron boundaries (Fig. 4c). Note that, despite the significant *p* value, the absolute number of overlapping events between ASB and GMAS is not large, possibly reflecting limited power in identifying either type of events and existence of many more ASB or GMAS events than included here.

Similarly, we examined the overlap between ASB and splicing quantitative trait loci (sQTLs) of GTEx tissues^22^ or The Cancer Genome Atlas (TCGA) samples^23^ (see Methods). Due to genotype difference between these samples and ENCODE cell lines, for each GTEx or TCGA sample, we only considered ASB SNVs that were annotated as heterozygous in its genotype. Other intragenic and heterozygous SNVs in the same sample were used as controls. Significant overlaps were observed for all GTEx tissues where sQTL data were available (Fig. 4d). For the TCGA data, we focused on samples of liver hepatocellular carcinoma (LIHC) and acute myeloid leukemia (LAML) to correspond to HepG2 and K562 cells, respectively. A significant overlap was observed in the LIHC samples (Supplementary Figure 13), but no ASB SNVs were observed in sQTL-associated exons or introns in the LAML samples. This result may be due to the relatively small number of sQTL-associated exons for LAML (1347, compared to 4387 for LIHC). Overall, our observations support the hypothesis that splicing factor-associated ASB imposes *cis*-regulation to splicing.

### Experimental validation of ASB SNVs for splicing regulation

Based on the above results, it is very likely that ASB SNVs of splicing factors are causal GVs responsible for genetic regulation of alternative splicing. To provide experimental support for this hypothesis, we tested five ASB SNVs regarding their potential impact on alternative splicing. These events were chosen to include ASB SNVs located in exons or within 500 nt away from exons. For each ASB SNV, the relevant exonic and intronic regions were cloned into a minigene reporter^6^ (see Methods). Two minigenes were created for each SNV, harboring the two alternative alleles, respectively. Upon transfection into HeLa cells, splicing of the middle exon was analyzed using reverse transcription PCR (RT-PCR) with primers targeting the flanking exons (Fig. 4e). All five exons were confirmed to have allele-specific splicing patterns.

The direction of splicing enhancement or repression by each allele depends on the binding preference and the functional roles of the associated RBP at specific target sites. For example, U2AF2 is known to enable splicing by recruiting the small nuclear ribonucleoprotein U2 and interacting with other spliceosomal components to define the 3′ splice site^24–26^. In the *LARS* RNA, we identified an ASB event of U2AF2 where the protein showed a binding preference for the C allele. Consistent with the expected function of U2AF2, the C allele in the minigene reporter was found to cause increased exon inclusion than the U allele. Similarly, both PRPF8 and SF3B4 are important players enabling spliceosome assembly^27,28^. Consistently, the preferred alleles of these proteins were associated with increased splicing (i.e., increased exon inclusion in *MAP2K3* or reduced intron retention in *OTUB1* and *IL17RB*). The experimental validations strongly support the functional roles of these ASB SNVs.

### ASB SNVs of UPF1 in 3′-UTRs may regulate RNA abundance

**Fig. 5:**
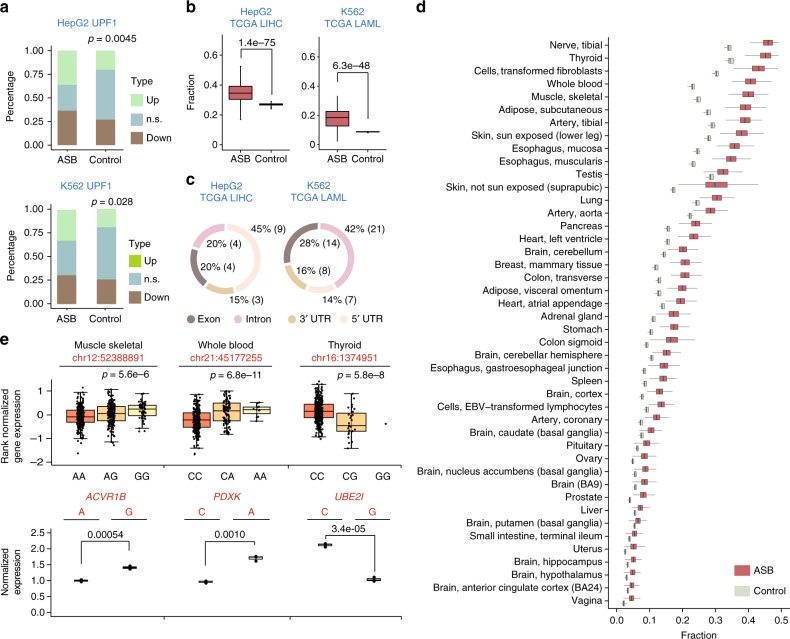
Functional relevance of allele-specific binding (ASB) single-nucleotide variants (SNVs) in regulating messenger RNA (mRNA) abundance. **a** Fraction of differentially expressed genes (up- or down-regulated, false discovery rate (FDR) <10%) upon UPF1 knockdown in HepG2 or K562 cells. n.s.: not significant. Data for genes with ASB SNVs of UPF1 in their 3′-untranslated regions (3′-UTRs) and control genes are shown, where the controls were chosen as genes without UPF1 enhanced crosslinking and immunoprecipitation (eCLIP) peaks and with similar expression levels as UPF1 targets (within +/−30% of RPKM). *P* values were calculated to test the null hypothesis that UPF1 ASB target genes are not enriched with up-regulated expression upon UPF1 knockdown (KD), compared to controls (binomial test). **b** Fraction of ASB SNVs located in expression quantitative trait loci (eQTL) genes among the union of ASB SNVs of all RNA-binding proteins (RBPs) of each cell line. eQTL genes were extracted from the The Cancer Genome Atlas (TCGA) project for liver hepatocellular carcinoma (LIHC) and acute myeloid leukemia (LAML), respectively, to match the cell type of HepG2 and K562. This fraction was calculated for each sample individually, with the distribution of all samples shown in the box plots. Control: fraction of control SNVs located in eQTL genes where control SNVs were randomly chosen from heterozygous SNVs located within genes. *P* values were calculated by pair-wise *t-*test. **c** Genomic context of ASB SNVs that are heterozygous in TCGA samples and located in the eQTL genes. Exon: coding exons; no non-coding transcripts existed among eQTL genes included in this analysis. **d** Similar as (**b**), for eQTL genes in GTEx tissues. **e** Expression of minigenes carrying alternative alleles of ASB SNVs in the 3′-UTR of mCherry. mCherry expression measured via real-time quantitative reverse transcription PCR (qRT-PCR) was normalized by that of eYFP (driven by bi-directional promoters). Three biological replicates were analyzed. *P* values were calculated by Student’s *t*-test. Box plots (top) show the normalized gene expression values of the host genes in selected GTEx tissues, grouped by genotypes of the ASB SNV (coordinates shown above box plots). The expression values and eQTL *p* values were obtained from the GTEx portal^33^. Boxplot center lines indicate the median and the boxes extend to lower and upper quartiles with whiskers depicting 1.5 interquartile range (IQR). (Source data are provided as a Source Data file)

Many RBPs regulate RNA abundance by binding to *cis*-regulatory elements in 3′-UTRs^29^. Among all RBPs with ASB events in 3′-UTRs, UPF1 had the highest number of events in both cell lines (Supplementary Figure 14a). Given UPF1’s well-known function in RNA degradation^16^, we asked whether genes with these ASB events demonstrated expression changes upon UPF1 KD. Using ENCODE RNA-Seq data sets, we analyzed differential expression of genes with UPF1 ASB in their 3′-UTRs (see Methods). Figure 5a shows the fractions of differentially expressed genes of these UPF1 targets. Compared to random controls, the UPF1 ASB targets are enriched with up-regulated genes upon UPF1 KD. The results support the hypothesis that UPF1 affects RNA abundance of its ASB target genes. Thus, it is likely that these ASB SNVs are functional variants in mediating RNA abundance through UPF1.

### ASB SNVs are significantly enriched in genes with eQTL

To further examine the association of ASB with *cis*-regulation of gene expression, we analyzed the enrichment of ASB-associated SNVs in genes with known expression QTL (eQTLs). Similarly as in the sQTL analysis, we obtained eQTL and genotype data of 376 LIHC and 200 LAML tumor sampls to correspond to HepG2 and K562 cells, respectively, generated by the TCGA project^30^. Next, we asked whether ASB SNVs are more enriched in eQTL genes than expected by chance. Similarly as in the splicing-related analysis, we only considered ASB SNVs that were annotated as heterozygous in the genotype of each sample. Other intragenic and heterozygous SNVs in the same sample were used as controls. As shown in Fig. 5b, across all samples, the fraction of ASB SNVs located in eQTL genes is generally higher than that of the control SNVs. Note that this analysis was not restricted to SNVs in 3′-UTRs since *cis*-elements in other regions may also directly or indirectly affect RNA abundance^31,32^. Indeed, we observed that ASB SNVs in eQTL genes are most often located in 5′-UTRs and introns in LIHC and LAML, respectively (Fig. 5c), the specific mechanisms of which should be examined in the future.

A similar analysis was carried out using eQTL data generated by the GTEx project^33^. For all the tissues included in this analysis, the occurrence frequency of ASB SNVs in eQTL genes was significantly higher than that in controls (Fig. 5d). It should be noted that since ASB patterns were identified in HepG2 and K562 cells, the observed overlap with eQTL genes here may be an underestimate of the actual overlap, given possible existence of cell-type specificity for certain ASB events. Therefore, the results here strongly support that ASB of RBPs likely impose regulation on mRNA abundance.

### Experimental validation of ASB SNVs regulating RNA abundance

To experimentally test the roles of ASB SNVs in regulating RNA abundance, we carried out reporter assays for three events randomly chosen from all ASB SNVs that are also eQTL variants in the GTEx data (Fig. 5e). The reporter has a bi-directional promoter that drives the expression of mCherry and eYFP (see Methods). Regions of the 3′-UTRs flanking the ASB SNVs were cloned as the 3′-UTR of mCherry (Methods), while eYFP serves as an internal control for gene expression. For each SNV, two versions of the reporter were constructed carrying each of the two alternative alleles. Upon transfection to HeLa cells, we measured mCherry and eYFP expression via real-time quantitative reverse transcription PCR (qRT-PCR). As shown in Fig. 5e, all three SNVs were confirmed as causal differential RNA abundance of the reporter. In addition, the observed allelic bias in expression is consistent with that observed in eQTL analysis by GTEx. Thus, these experiments confirmed the functional roles of these ASB SNVs in modulating RNA abundance.

### ASB SNVs overlap disease-associated GVs

**Fig. 6:**
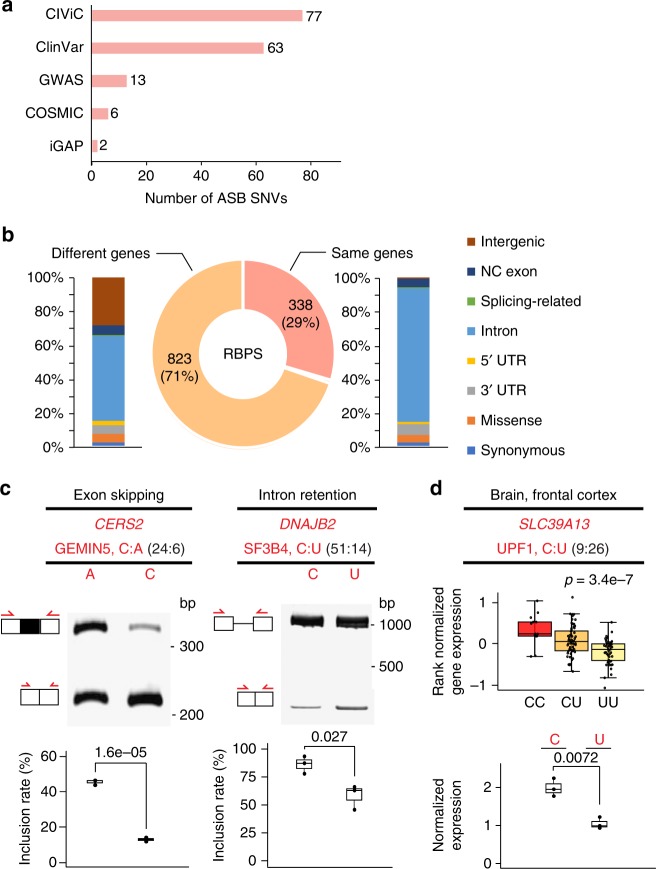
Allele-specific binding (ASB) events inform functional interpretation of disease-associated variants. **a** Numbers of ASB single-nucleotide variants (SNVs) that are also disease-related SNVs annotated by different databases. **b** Genomic context of genome-wide association study (GWAS) single-nucleotide polymorphisms (SNPs) (stacked bars) located in the same or different genes as ASB SNVs. Most GWAS SNPs are located in introns, whose function was elusive. NC exon: exons in non-coding transcripts. Splicing-related: those located in splice site signals. **c** Splicing reporter validation of two ASB SNVs, similar as Fig. 4e. (Images are cropped, with uncropped images in Supplementary Figure 16.) **d** Minigene reporter validation of one SNP for its function in modulating messenger RNA (mRNA) abundance, similar as Fig. 5e. Boxplot center lines indicate the median and the boxes extend to lower and upper quartiles with whiskers depicting 1.5 inter-quartile range (IQR). (Source data are provided as a Source Data file)

To examine whether ASB by RBPs may explain the functional roles of disease-related GVs, we compared ASB SNVs in this study with known disease-associated GVs included in several databases: GWAS (genome-wide association study), COSMIC, ClinVar, CIViC, and iGAP. As shown in Fig. 6a, a total of 154 unique ASB SNVs have known disease relevance according to this analysis. In addition, we asked whether ASB SNVs were in linkage disequilibrium (LD) with single-nucleotide polymorphisms (SNPs) reported in GWAS (LD defined as *D′* > 0.9 and *r*^2^ > 0.8, and within 200 kb in distance, see Methods for details). A total of 1676 ASB SNVs (34.7% of the 4825 ASB SNVs in total combining data from HepG2 and K562) were in LD with 1161 GWAS SNPs (Fig. 6b). This high percentage of ASB SNVs with GWAS association supports the potential functional relevance of ASB. Among these GWAS SNPs, 29% were in the same genes as the ASB SNVs (Fig. 6b). The vast majority of the GWAS SNPs were located in introns whose functional consequence had been hard to predict.

We experimentally tested the functional impacts of three disease-associated ASB events. Specifically, two events were chosen randomly as candidates that may alter splicing by requiring the SNVs to reside in exons or within 500 nt to exon–intron boundaries. The splicing reporters were constructed similarly as described above. As shown in Fig. 6c, both SNVs were confirmed as splicing-altering variants. Specifically, The SNP rs267738 is associated with multiple traits in GWAS, such as the blood protein level and rhegmatogenous retinal detachment^34,35^. It demonstrated ASB pattern in the eCLIP data of GEMIN5, a small nuclear RNA-binding component of the survival of motor neurons complex^36^. This SNP is located in the gene *CERS2* with known functional relevance in cancer^37,38^. Interestingly, the SNP was annotated as a missense variant in the GWAS catalog. Our data demonstrated significant splicing changes caused by this SNP, suggesting that exonic SNVs that appear to be nonsynonymous could function by altering splicing, an aspect that has been largely overlooked. Note that GEMIN5 may affect splicing in either direction (enhancing or repressing)^39^. In our experiment, the GEMIN5-preferred allele (C) was associated with increased exon skipping compared to the alternative allele A. Through GEMIN5 KD experiments, we confirmed that the C allele (but not the A allele) responded to KD significantly (higher exon inclusion upon KD) (Supplementary Figure 15).

The SNP rs3731896 in the gene *DNAJB2* was identified as an ASB SNV of the RBP SF3B4. It is a known GWAS SNP associated with educational attainment^40^. In the reporter assays, we observed that this SNP alters intron retention of its host intron. *SF3B4* encodes for a subunit of the splicing factor SF3B^41^ that is part of the spliceosomal complex. It is generally expected that SF3B4 enables spliceosome formation. However, we observed that the C allele (preferred binding by SF3B4) is associated with increased intron retention. The result indicates that the function of SF3B4 may be more complex than expected, consistent with a previous observation that reduced expression of this gene was associated with a predominant reduction in intron retention^42^.

The SNP rs2293577 is located in the 3′-UTR of the gene *SLC39A13* that is annotated to be associated with Alzheimer’s disease by the iGAP consortium^43^. The U allele of this SNP in the RNA is preferred for UPF1 binding. We chose to validate the function of this SNP in altering mRNA abundance using the bi-directional mCherry/eYFP reporters as described above. Consistent with the expected function of UPF1 in RNA degradation and GTEx eQTL results (Fig. 6d), the U allele was associated with reduced RNA expression. Intriguingly, the region harboring this SNP is also associated with an intron retention event (Supplementary Figure 14b). Thus, we also tested the impact of this SNP on intron retention using the splicing reporter. The results confirmed that the U allele in the RNA caused a significant level of retained introns compared to the C allele (Supplementary Figure 14b). These results suggest that the SNP likely imposes multiple types of functional impacts on *SLC39A13* expression.

## Discussion

In this study, we developed a new method called BEAPR to identify ASB events using the eCLIP-Seq data. eCLIP-Seq captures transcriptome-wide protein–RNA interaction profiles. Compared to previous CLIP methods, eCLIP improves the efficiency and reproducibility in library generation and yields high usable read percentages across diverse RBPs^8^. The large number of eCLIP-Seq data sets made available by the ENCODE project, with biological replicates and paired size-matched input controls, affords a unique opportunity to examine protein-RNA interaction in an allele-specific manner.

Quantitative analysis of SNVs in CLIP reads is challenging in that the read coverage of a single nucleotide is relatively low compared to that in an average RNA-Seq data set. In addition, technical biases, such as those due to crosslinking, may confound the estimated allelic bias and lead to false-positive ASB predictions. BEAPR addresses these potential issues by modeling read count variability and crosslinking bias and filtering for other possible technical biases. Using simulated reads, we demonstrated that BEAPR outperforms standard methods for read count comparisons. Importantly, we observed that the other methods suffered from overdispersion in both simulated and actual eCLIP data, which led to their high false-positive rates. In contrast, BEAPR is robust to the variance in the input read count data and overcomes the overdispersion issue.

BEAPR identified thousands of ASB events using the ENCODE eCLIP data. Supported by experimental validations, we demonstrated that the ASB patterns helped to inform functional predictions of SNVs in regulating alternative splicing and mRNA abundance. The majority of these SNVs are located in the introns or 3′-UTRs. Thus, these results helped to address one of the most challenging questions in the post-genomic era: the functional relevance of non-coding variants in human genomes.

It is increasingly appreciated that non-coding variants may affect post-transcriptional gene regulation and many contribute to disease-related processes^3^. For example, it was estimated that 35% of disease-causing point mutations disrupt splicing by altering splice site signals or auxiliary regulatory elements in the exons or introns^3^. Despite the widely recognized importance, systematic prediction of causal GVs that alter post-transcriptional gene regulation has been a major challenge. Most methods, such as splicing or gene eQTL analyses, rely on detection of correlative relationships without the capacity to pinpoint the exact causal GVs. ASB of RBPs provides a direct means to examine the function of SNVs. The ASB-based analyses presented in this study demonstrated that it is highly feasible to employ this approach for causal SNV detection in post-transcriptional regulation. Nonetheless, it should be clarified that, similarly as the fact that protein–RNA binding alone does not always lead to a functional consequence, existence of ASB alone does not always entail allele-specific function of the protein. The ASB data should be combined with other molecular phenotypes (such as splicing or gene expression level) to interpret the function of SNVs.

In addition to splicing and mRNA abundance, ASB patterns may help to identify functional SNVs involved in other aspects of RNA metabolism, such as RNA localization or RNA secondary structures. The specific function of SNVs should depend on the roles of the RBPs demonstrating ASB patterns. Therefore, we expect that allele-specific analyses of eCLIP will be an essential approach to deciphering the function of non-coding variants in the RNA.

## Methods

### Preprocessing of the ENCODE eCLIP data

eCLIP data sets generated from the HepG2 and K562 cell lines were downloaded from the ENCODE data portal. Raw reads were demultiplexed, adapter-trimmed, and mapped according to established eCLIP data processing procedures of the ENCODE project^9^. After removal of PCR duplicates, the remaining uniquely mapped reads were called “usable” reads for ASB analysis. eCLIP peaks were identified using read 2 (*R*_2_) of the paired-end reads via CLIPper^13^, with options -s hg19 -o -bonferroni -superlocal-threshold-method binomial-save-pickle. In this work, eCLIP peaks were retained for subsequent analyses if the library-normalized read coverage in at least one replicate is ≥4-fold of that in the corresponding region in the SMInput.

### Identification of crosslinking sites in eCLIP peaks

To examine the potential existence of crosslinking bias, BEAPR first determines crosslinking sites within eCLIP peaks. In eCLIP assays, reverse transcription of complementary DNA (cDNA) usually terminates at the protein–RNA crosslinking site^8^. Thus, a crosslinking site is expected to coincide with the start position of a significant number of *R*_2_ reads compared to random expectations. It should be noted that if a protein binds to a cluster of motifs, multiple crosslinking sites may be identified within one eCLIP peak. This is because that reverse transcription may sometimes read-through a 3′ crosslinking site and reach the upstream ones.

To identify crosslinking sites, we carried out a similar procedure as in our previous work^8^. Specifically, for each eCLIP peak, the 5′ end positions of usable *R*_2_ reads within the peak were identified. For each nucleotide position *i* in the peak, the number of *R*_2_ reads, *m*_*i*_, whose 5′ end coincides with position *i* was obtained. The actual *m*_*i*_ values were compared to random expectations obtained by permuting the positions of eCLIP reads within a peak. Empirical FDR was calculated and a minimum FDR of 0.001 was used to call crosslinking sites.

### Normalization of allelic read counts by crosslinking bias

To identify inherent crosslinking bias for an eCLIP experiment, BEAPR calculates the relative abundance of the four nucleotides at each position flanking the crosslinking sites in the SMInput sample (Fig. 1b). The observed relative sequence bias in the SMInput is unlikely resulted from the binding preference of specific RBPs. This bias is thus referred to as the crosslinking bias, which is estimated for each experiment. It is used to normalize the observed allele-specific read counts of SNVs in the vicinity of crosslinking sites.

Prior to read count normalization for each SNV, we removed low-quality read bases by requiring a minimum Base Alignment Quality score of 10^44^. After this procedure, we examined the 51-nt region flanking the crosslinking site in each eCLIP peak. For each offset position *d*, −25 ≤ *d* ≤ 25, relative to the crosslinking site, the crosslinking bias *q*(*a, d*) of the nucleotide *a* at the offset position *d* was calculated as described above. Let *y*_*i,a,j*_ be the number of eCLIP reads mapped to the allele *a* at the SNV *i* in the eCLIP replicate *j*. *R*_*j*_ denotes the total number (in millions) of eCLIP reads in the replicate. The normalize read count *x*_*i,a,j*_ of *y*_*i,a,j*_ was calculated as $x_{i,a,j} = y_{i,a,j} \times \left( {q\left( {a,d} \right) \times R_j} \right)^{ - 1}$. If *d* > 25 or *d* < −25, *q*(*a, d*) was set to be 0.25.

### Estimation of expected variance of normalized read counts

For each allele *r* at a heterozygous SNV *i*, let the variance of the normalized read counts across the eCLIP replicates be $\sigma _{r,i}^2$. Since only a small number of replicates are available, the sample variance $\sigma _{r,i}^2$ is a poor estimator of the expected variance of the normalized read counts for the allele *r*. Hence, we developed the following procedure that considers allelic read counts from all SNVs to enhance the estimation of the expected variance. Specifically, for each allele *r* at an SNV *i* located in an eCLIP peak, we calculated the mean *μ*_*r,i*_ and variance $\sigma _{r,i}^2$ of its normalized read counts across the CLIP replicates. Using each pair of the mean and variance values, *μ*_*r,i*_ and $\sigma _{r,i}^2$, we calculated the square of the coefficient of variation (CV2) value, *ω*_*r,i*_. Then, a LOESS regression function was applied to fit all *ω*_*r,i*_ and log_2_(*μ*_*r,i*_) values, where the CV2 was the response variable and the log 2-scaled mean was the explanatory variable (Fig. 1c). To predict the expected variance of the normalized read counts for an allele *r*’, let $\hat \omega _{r\prime ,i}$ be the CV2 value moderated by the LOESS regression function. The expected variance $\hat \sigma _{r\prime ,i}^2$ for the allele *r'* at an SNV *i* was calculated as $\hat \sigma _{r\prime ,i}^2 = \hat \omega _{r\prime ,i} \times \mu _{r\prime ,i}^2$.

### Identification of ASB events

Let *r* and *a* denote the reference and alternative allele at an SNV site *i* and $X_{A,i} = \left\{ {x_{A,i,1},...,x_{A,i,k}} \right\}$ be the normalized read counts for an allele *A* at the SNV *i* in all the *k* CLIP replicates. Our null hypothesis is that there is no ASB at the SNP *i* such that *r* and *a* are equivalently represented in the associated eCLIP peak. To test the null hypothesis, we examined whether *μ*_*r,i*_ = *μ*_*a,i*_ given *X*_*r,i*_ and *X*_*a,i*_. An alternative method to test the difference of the two mean values is *t*-test. However, due to the small sample size of the allelic read counts from individual genomic features, *t-*test was shown to be inapplicable to genomic read counts data derived from a limit number of replicates^45^. Hence, we derived the following empirical Gaussian distribution, which can adapt to different types of read count data with high or low dispersion heterogeneity^45^, to model the normalized read counts. A novelty of our method is the incorporation of the global mean-variance relationship moderated by the LOESS regression into the statistical test for the equivalence of the two mean values.

We assume

1 $$X_{A,i} \sim {\cal N}\left( {\mu ,\sigma ^2} \right),$$

where *μ* is the mean and *σ*^2^ the variance of the Gaussian distribution. In addition, we assume the prior distribution of *μ* follows a normal distribution:

2 $$\mu \sim {\cal N}\left( {\mu _0,\sigma _0^2} \right),$$

where the mean *μ*_0_ and the variance $\sigma _0^2$ are hyper-parameters.

At an SNV *i*, an allele was named the major allele, denoted as *M*, if its average allelic read counts across the CLIP replicates was higher than that of the other allele. Otherwise, the allele was called the minor allele, denoted as *m*. In BEAPR, we calculate the empirical probability $P\left( {\mu = \mu _{m,i}|X = X_{M,i}} \right)$ that the average read count of the minor allele, *μ*_*m,i*_, was generated from the same distribution from which the normalized read counts for the major allele *M* was observed. Thus, the *p* value ${\cal P}$ to reject the null hypothesis was defined as:

3 $${\cal P} = 2 \times {\int \nolimits_{ - \infty }^{\mu _{m,i}}} P\left( {\mu |X_{M,i}} \right){\mathrm{d}}\mu.$$

Based on Bayes rule, $P\left( {\mu |X_{M,i}} \right) \propto P\left( {X_{M,i}|\mu } \right)P(u)$. Assume the expected variance for the allele *M* is $\hat \sigma _{M,i}^2$, which is estimated as described in the last section. By combining the empirical probability *P*(*μ*|*X* = *X*_*M,i*_) with the distributions of *X* and *μ*, the empirical probability can be rewritten as a Gaussian distribution such that $P\left( {\mu |X = X_{M,i}} \right) = {\cal N}\left( {\mu |\tilde \mu ,\tilde \sigma ^2} \right)$, where the mean $\tilde \mu$ is:

4 $$\tilde \mu = k\hat \sigma _{M,i}^{ - 2}\left( {\sigma _0^{ - 2} + k\hat \sigma _{M,i}^{ - 2}} \right)^{ - 1}\mu _{M,i} + \sigma _0^{ - 2}\left( {\sigma _0^{ - 2} + k\hat \sigma _{M,i}^{ - 2}} \right)^{ - 1}\mu _0,$$

where *k* is the number of eCLIP replicates, and the variance $\tilde \sigma ^2$ is:

5 $$\tilde \sigma ^2 = \left( {\sigma _0^{ - 2} + k\hat \sigma _{M,i}^{ - 2}} \right)^{ - 1}.$$

Since the prior probability of *μ* is unknown, we assumed that $\sigma _0^2 \to \infty$. Thus, the posterior probability to observe the mean *μ*_*m,i*_ for the minor allele *m* given *X*_*M,i*_ is:

6 $$P\left( {\mu |X_{M,i}} \right) \sim {\cal N}\left( {\mu |\mu _{M,i},\hat \sigma _{M,i}^2k^{ - 1}} \right).$$

To adjust for multiple testing, FDRs were calculated. A minimum FDR of 10% was required to call significant ASB events in the ENCODE data sets.

### Identification of heterozygous SNVs in eCLIP reads

BEAPR includes a procedure to call heterozygous SNVs directly from the eCLIP data. As candidate SNVs, we obtained known SNPs or mutations from various databases, including dbSNP, GTEx, TCGA, ExAC, and COSMIC. For each cell line, we pooled all the SMInput data sets together and calculated the allelic read counts at all candidate SNV locations. A candidate SNV was predicted as a heterozygous SNV in the cell line if the total read coverage was at least 10 and the allelic ratio of the reference allele was between 0.25 and 0.75. In addition, we used whole-genome DNA sequencing data of the two cell lines to identify heterozygous SNVs using the same method as in our previous work^14^. It should be noted that in the analysis of ASB SNVs, heterozygous SNVs outside eCLIP peaks were discarded. However, the identification of heterozygous SNVs can be carried out for any region satisfying the above read coverage requirement. The number of heterozygous SNVs identified in the two cell lines with or without the eCLIP peak filter is shown in Supplementary Figure 5b.

### Simulation of allele-specific read counts

To simulate allele-specific read counts that mimic those in actual eCLIP data sets, we used the eCLIP data of SRSF1 in the K562 cell line generated by ENCODE. eCLIP peaks were identified as described above. For heterozygous SNPs (dbSNP 144) located in the peaks, we obtained their total read coverage in each replicate. The empirical total read coverage distributions were used to generate independent sets of simulated read counts. For each simulated SNP, its total read coverage was sampled from the above distribution. Its allelic ratio was set to be 0.5, unless it is a simulated ASB SNP (with allelic ratio being 0.7, 0.8 or 0.9). The allelic read counts for each SNP were determined using a zero-truncated negative binomial distribution with the expected variance set to be equivalent to the observed variance between the two replicates of SRSF1 eCLIP as a function of total read coverage.

To add crosslinking-induced bias, the simulated allelic read counts for each SNP was rescaled based on the sequence propensity estimated from the SRSF1 data (similar as Fig. 1b), for SNPs located within 25 nt of crosslinking sites. To simulate additional SNPs with high variances in their read counts, we required the standard deviation of the SNP read counts between the two replicates be greater than that of 95% of SNPs in the SRSF1 data.

### Evaluation of performance

Using simulated read counts, the overall performance of ASB prediction was evaluated in terms of precision, TP × (TP + FP)^−1^, and recall, TP × (TP + FN)^−1^, where TP is the number of true positives, FP is the number of false positives, and FN is the number of false negatives. The area under the precision-recall curve (AUC) was calculated. We used this AUC value instead of that of a receiver operating characteristic (ROC) curve, because the number of SNVs with ASB or not were extremely unbalanced. A recent study suggested that precision-recall curves were more informative than ROC curves on unbalanced data^46^. Moreover, we also evaluated the prediction methods by sensitivity (SEN), TP × (TP + FN)^−1^, and specificity (SPE), FP × (TN + FP)^−1^, where *TN* is the number of true negatives. We reported sensitivity at 95% specificity (SEN95) and specificity at 95% sensitivity (SPE95) as additional performance metrics to assess the methods.

### Posterior filters for quality control in BEAPR

In applying BEAPR to actual eCLIP-Seq data, we incorporated a number of posterior filters to ensure the quality of the predicted ASB events. Note that no such filters were applied to simulated data. First, since ASB analysis handles single nucleotides in sequencing reads, it suffers from similar problems as in RNA-editing analysis. Thus, we implemented posterior filters that are similar as those widely used in identifying RNA-editing sites^47^. Specifically, predicted ASB candidates were excluded if they are located in (1) microsatellites annotated by RepeatMasker^48^ (downloaded from UCSC genome browser), (2) homopolymeric pentamers, or (3) regions with mapping ambiguity. For the last filter, we used BLAT^49^ to align the 101 nucleotide sequence centered at each ASB SNV. ASB candidates in sequences with ≥95% similarity with other genomic regions were discarded. All the above filters are implemented in the BEPAR pipeline.

Allelic imbalance in eCLIP reads may be observed as a result of allele-specific expression (ASE) of the host gene, regulated by gene-level control mechanisms, such as allele-specific transcriptional activation. Such allelic bias may be identified as ASB in eCLIP data, but does not reflect bona fide allelic binding events of any RBPs. Using RNA-Seq data of control HepG2 and K562 cells in the ENCODE project, we identified genes that showed whole-gene level ASE using ASARP^21^. In this analysis, we focused on genes that had at least two heterozygous SNVs each with a minimum of 10 reads (defined as testable SNVs). Genes whose testable SNVs all demonstrated allelic bias (FDR < 0.05) was deemed as ASE genes. If a gene was detected as an ASE gene in at least two RNA-Seq data sets (out of 24 total), we further required that the reference allele frequency of all SNVs in this gene to be <40% or >60% based on RNA-Seq reads. ASB candidates in such genes were discarded.

In read alignment of SNVs using a reference genome, it is known that mapping bias that favors the reference alleles may exist^21^. To account for such possible bias, we calculated the distribution of allelic ratios at known heterozygous SNVs in the SMInput data. The mean *θ* and standard deviation *σ* of this distribution were calculated. For each ASB candidate *i* whose allelic ratio was *θ*_*i*_, we calculated a *Z*-score, $z_i = \left\| {\theta _i} \right. - \theta\left\| \right. \times \sigma ^{ - 1}$, to evaluate how likely the ASB observation was due to reference mapping bias. ASB candidates were discarded if the *Z*-scores were smaller than 1.0.

Empirically, we observed high densities of eCLIP peaks from multiple RBPs exist in a small number of genomic regions. This observation may reflect artifacts in eCLIP that generated “hotspot” IP regions. To alleviate the impact of this possible artifact, we binned the genome into non-overlapping windows of 2000 nucleotides and calculated the number of the ASB candidates shared by more than four RBPs in each window. We identified a small number of windows in the two cell lines where the ASB candidates shared by more than four RBPs significantly outnumbered those in the other windows, such as chr14:24,610,000–24,612,000 in HepG2, chr17:41,466,000-41,468,000 in K562, and chr19:34,882,000–34,884,000 in both cell lines. We thus discarded ASB candidates in these genomic regions.

The number and percentage of ASB events removed by each stepwise filter are shown in Supplementary Figure 7a.

### Motif analysis

The position-specific motif enrichment plots (Fig. 3) were generated as follows. Within each peak harboring an ASB event, k-mer occurrence at each position flanking the ASB SNV was counted. Note that at the ASB SNV, the sequence of the major allele in eCLIP was used. The k-mers used here are pentamers identified by RBNS for each RBP^9^. The frequency of the RBNS pentamers in all ASB regions of an RBP was calculated. As controls, we randomly picked a genomic sequence to match each region with ASB in terms of the type of region (e.g., intron, 3′-UTR etc) and GC content (±10%). A total of 10 random sets of sequences were selected, with each set containing the same number of sequences as the number of ASB regions of an RBP. The fold enrichment of RBNS pentamers in ASB regions relative to the random controls was calculated and visualized in Fig. 3. The location of ASB SNVs relative to the sequence motifs of each RBP (Fig. 3) was determined using MOODS (*p* < 0.05)^50^.

### Splicing-related analysis of RNA-Seq data

RNA-Seq data of RBP KD or associated controls were obtained from the ENCODE portal. The PSI values of annotated exons (Gencode (basic v24) annotation) were calculated using inclusion and exclusion reads of the alternative regions^6^. GMAS events in HepG2 and K562 cells (with control short hairpin RNA (shRNA) transfections) were identified using ASARP^6,21^. To identify overlap between an ASB SNV and a GMAS exon, the ASB SNV was required to reside in the GMAS exon or within 500 nt from exon–intron boundaries. We tested the statistical significance of the overlap via the hypergeometric test, where the background is the total number of heterozygous SNVs in genes harboring ASB SNVs.

### Overlap between ASB events and QTL-related exons and genes

In the analysis of GTEx data, we obtained genotype data for 515 human donors (GTEx v6p release), from which heterozygous SNPs were identified for each individual. QTL-related exons were defined as those whose junctions were associated with GVs significantly (FDR < 0.05), in the GTEx Pilot analysis^22^. Genes with eQTL were extracted from GTEx v6p release^33^.

For the TCGA samples, genotype data of 200 LAML and 376 LIHC tumor samples were obtained from the TCGA data portal (https://tcga-data.nci.nih.gov/tcga/). QTL-related exons were defined as those associated with splicing QTL in the CancerSplicingQTL database^23^. Genes with eQTL were extracted from the PancanQTL database^30^.

### Comparison of ASB SNVs and GWAS SNPs

To identify GWAS SNPs in LD with ASB SNVs, the GWAS catalog was downloaded from the NHGRI GWAS page at http://www.genome.gov/gwastudies/ on December 17, 2014. LD data of the CEPH (Utah residents with ancestry from northern and western Europe) population were obtained from the International HapMap Project^51^. In our GWAS LD analysis, an ASB SNV is defined as in LD with a GWAS SNP if both variants were located in the same LD block that passed the thresholds *D*′ > 0.9 and *r*^2^ > 0.8. Additionally, the distance between the GMAS SNV and GWAS SNP was required to be <200 kb.

### RNA-Seq data analysis of UPF1 KD

RNA-Seq data of UPF1 KD and associated controls were obtained from the ENCODE portal. Differential gene expression analysis was carried out using DESeq2^52^ with an FDR cutoff of 10%. The Gencode (basic v24) gene annotation was used for this purpose.

### Minigene reporters for splicing assays

For exon skipping events, the candidate exon and ~400 nt upstream and downstream flanking introns were amplified using HeLa or K562 genomic DNA. After double digestion by *Hind*III and *Sac*II or *Eco*RI and *Sac*II, the DNA fragments were sub-cloned into pZW1 splicing reporter plasmids^53^. For intron retention events, the candidate intron and its flanking exons were cloned into the pcDNA3.1 plasmids. Final constructs were sequenced to ensure that a pair of plasmids containing the two alternative alleles of the SNV was obtained.

### Transfection, RNA extraction, reverse transcription, and PCR

Minigene constructs were transfected into >90% confluence HeLa cells (ATCC, CCL-2) using Lipofectamine 3000 (Thermo Fisher Scientific, L300015). Cells were harvested 24 h post transfection and total RNA was isolated using TRIzol (Thermo Fisher Scientific, 15596018), followed by Direct-zol RNA Mini prep (Zymo Research, R2072). cDNA was prepared from 2 μg of total RNA by SuperScript IV First-Strand Synthesis System (Thermo Fisher Scientific, 18091050) and one-twentieth of the cDNA was used as template to amplify both inclusion and exclusion of the candidate exon by PCR of 28 cycles.

### Gel electrophoresis and quantification

Five microliter of PCR product was loaded onto 5% polyacrylamide gel and electrophoresis at 70 V for one and a half hours. The gel was then stained with SYBR^®^ Safe DNA Gel Stain (Thermo Fisher Scientific, S33102) for 30 min before imaging via Syngene SYBRsafe program (Syngene). The expression level of spliced isoforms was estimated using the ImageJ software (http://imagej.nih.gov/ij/). Inclusion or intron retention rate (% inclusion) of the target exon was calculated as the intensity ratio of upper × (upper + lower)^−1^ bands.

### Bi-directional reporter constructs for 3′-UTR analysis

To test the function of ASB events in 3′-UTRs, ~700–1000 nt of the 3′-UTR regions including the ASB SNV were amplified using genomic DNA extracted from HMLE, HeLa, or K562 cells. Site mutations were generated for alternative alleles for each SNV using overlap-extension PCR. After double digestion by *Cla*I and *Sal*I-HF, the DNA fragments were sub-cloned into the 3′-UTR of mCherry in the bi-directional reporter plasmid pTRE-BI-red/yellow that encodes for both mCherry and eYFP^54^. Final constructs were sequenced to ensure that a pair of plasmids containing the two alternative alleles of the SNV was obtained.

### Real-time PCR

The real-time PCR reaction was performed using SsoAdvanced Universal SYBR Green Supermix (Bio-Rad, 172-5270) and CFX96 Touch Real-Time PCR detection system (Bio-Rad) according to the manufacturer’s instructions. The mRNA expression level associated with each allele of the ASB SNV was measured by the mCherry expression levels, which was normalized against that of eYFP.

### Purification of recombinant human PTBP1

The human PTBP1-pET28a expression vector was a gift from Dr. Douglas Black. It was transformed into BL21 Star (DE3)-competent cells (Thermo Fisher Scientific, C602003). Protein induction was carried out via 1 mM isopropyl β-d-1-thiogalactopyranoside (IPTG) treatment in 50 mL cultured cells (OD = 0.8) for 16 h at 215 rpm at 28 °C. Next, cultured cells were centrifuged at 7000 × *g* for 5 min at 4 °C and the pellets were resuspended with ice-cold 5 mL lysis buffer (1× BugBuster, 20 mM sodium phosphate, pH 7.7, 500 mM NaCl, 20 mM imidazole, 1 mM dithiothreitol (DTT), 0.5× protease inhibitor cocktail, 100 μg/mL lysozyme, 100 U DNAse I). After 30 min incubation, the lysate was disrupted using three times sonication at 30% amplitude for 30 s with 1 s pulse. Subsequently, the lysate was centrifuged at 15,000 × *g* for 15 min at 4 °C. The supernatant was collected and filtered using 0.45 μm syringe filter. The sample was loaded into the HisTrap HP column (GE Healthcare, 17-5247-01) using Biologic LP system (Bio-Rad, 7318304) and washed with 20 mL buffer A (20 mM sodium phosphate, pH 7.7, 500 mM NaCl, 20 mM imidazole, 1 mM DTT). The sample was eluted with 500 mM imidazole in buffer A. Purity of the recombinant PTBP1 protein was determined by SimplyBlue SafeStain (Thermo Fisher Scientific, LC6060) and western blot using anti-HIS antibody (Santa Cruz Biotech, sc-8036, 1:500 dilution). Clean fractions (E28 and E33, Supplementary Figure 12) were combined (~3 mL). Salt and small size of non-specific proteins were removed by incubating in 20 K Slide-A-Lyzer dialysis cassette (Thermo Fisher Scientific, 66003) with 1 L Buffer A in a cold room overnight. Protein concentration was measured by Pierce Coomassie (Bradford) protein assay kit (Thermo Fisher Scientific, 23200) and Turner spectrophotometer SP-830.

### In vitro transcription of PTBP1 target RNA

ASB candidates overlapping with PTBP1 binding motif were selected and 100 μM of sense and antisense oligos including T7 promoter were annealed with oligo annealing buffer (10 mM Tris-HCl, pH 8.0, 1 mM EDTA, pH 8.0, 100 mM NaCl) at 95 °C for 5 min in a heat block and then cooled slowly to 28 °C for 2 h. In vitro transcription was performed using 1 μg of annealed oligos and HiScribe T7 high yield RNA synthesis kit. In vitro synthesized RNAs were treated with 10 U RNAse-free DNAse I (Thermo Fisher Scientific, EN0525) at room temperature for 30 min, and then purified by RNA Clean & Concentrator-5 Kit (Zymo Research, R1015). Next, RNA samples were treated with 10 U shrimp alkaline phosphatase (NEB, M0371S) at 37 °C for 1 h and then labeled with 0.4 μL of γ-^32^P-ATP (7000 Ci/mmol, MP Biomedicals) using 20 U T4 polynucleotide kinase (NEB, M0201S). Subsequently, RNA probes were purified using 5% urea-PAGE extraction and RNA Clean & Concentrator-5 Kit. RNA concentration was measured by Qubit 2.0 fluorometer (Thermo Fisher Scientific).

### Electrophoretic Mobility Shift Assay

The purified RNA probes (20 pmol) and recombinant PTBP1 protein (0, 0.6, 1.2, 2.5, and 5 μg) were incubated in 15 μL of buffer A (20 mM sodium phosphate, pH 7.7, 500 mM NaCl, 20 mM imidazole, 1 mM DTT, 0.1× protease inhibitor cocktail, 10 U RNAse inhibitor) at 28 °C for 30 min, and then loaded onto 5% TBE-PAGE and ran at 75 V for 1.5 h. The gel was processed without drying, covered with clear folder, and exposed to X-ray film at −80 °C.

### Lentivirus mediated GEMIN5 KD

pLKO1 non-target control-shRNA (SHC016) and GEMIN5-shRNA (TRCN0000147159, TRCN0000129034, TRCN0000150146, TRCN0000130416, TRCN0000149925) constructs were used for this experiment. Lentiviruses were produced as follows:^55^ pLKO1, pCMV-d8.91 and pVSV-G were co-transfected into HEK293T cells (ATCC, CRL-11268) using Lipofectamine 3000 (Thermo Fisher Scientific, L3000015). After 48 h co-transfection, viruses were harvested and used to infect HeLa cells with polybrene (8 μg/mL). After 24 h, cells were incubated with puromycin (2 μg/mL) for 3–7 days selection.

To examine the efficiency of GEMIN5 KD, cell lysates were prepared with RIPA buffer and used for Western blot with GEMIN5 (Bethyl Lab, A301-325A, Lot# A301-325A-1, 1:200 dilution) and β-actin antibody (Santa Cruz, sc-47778, Lot# J2915, 1:500 dilution). The splicing reporter assay for *CERS2* was performed as described above.

### Reporting Summary

Further information on experimental design is available in the Nature Research Reporting Summary linked to this article.

## Supplementary information

Supplementary Information

Reporting Summary

Description of Additional Supplementary Files

Supplementary Data 1

Supplementary Data 2

Source Data

## Data Availability

The BEAPR package is freely available at: https://github.com/gxiaolab/BEAPR/wiki. This software package was developed and tested on Linux with g + + 4.4.5 and R 3.2.3.

## Data availability

All data sets used in this study can be obtained from the ENCODE project website at http://www.encodeproject.org. The source data underlying the main figures are provided as a Source Data file. All other relevant data is available upon request.
