# Supplementary material for: Allele-specific binding of RNA-binding proteins reveals functional genetic variants in the RNA
Source: Nat Commun. 2019 Mar 22;10:1338. doi: 10.1038/s41467-019-09292-w (PMC6430814; doi:10.1038/s41467-019-09292-w)

Supplementary Information

Allele-specific binding of RNA-binding proteins reveals functional genetic variants in the RNA

Yang et al.

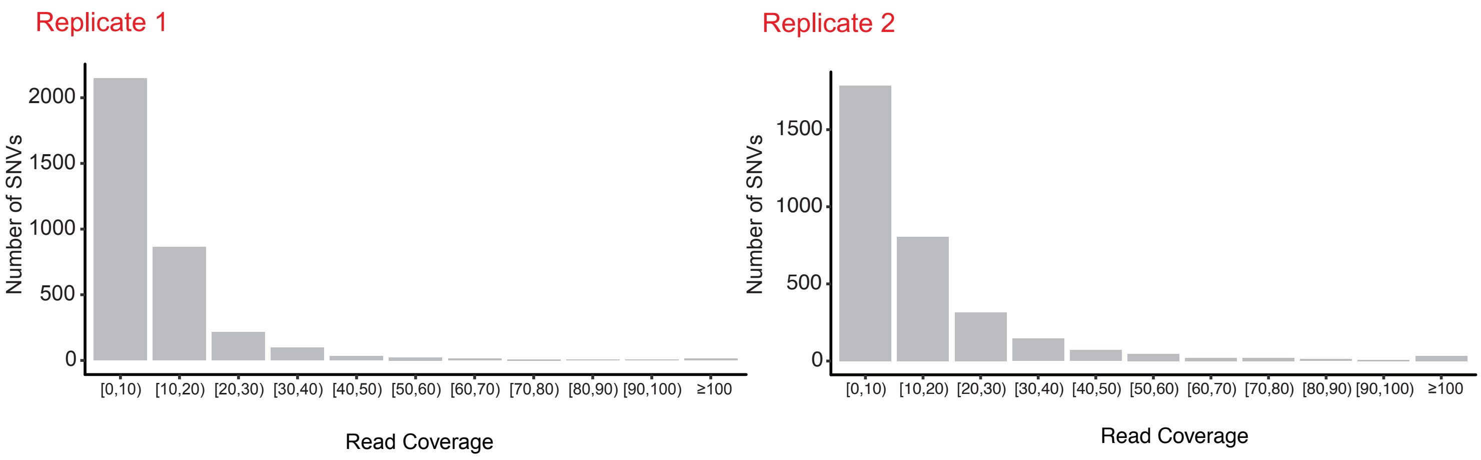

Supplementary Figure 1. Read coverage of known SNPs (dbSNP version 144) in the eCLIP-Seq peaks of SRSF1 in K562 cells. Two replicates are shown.

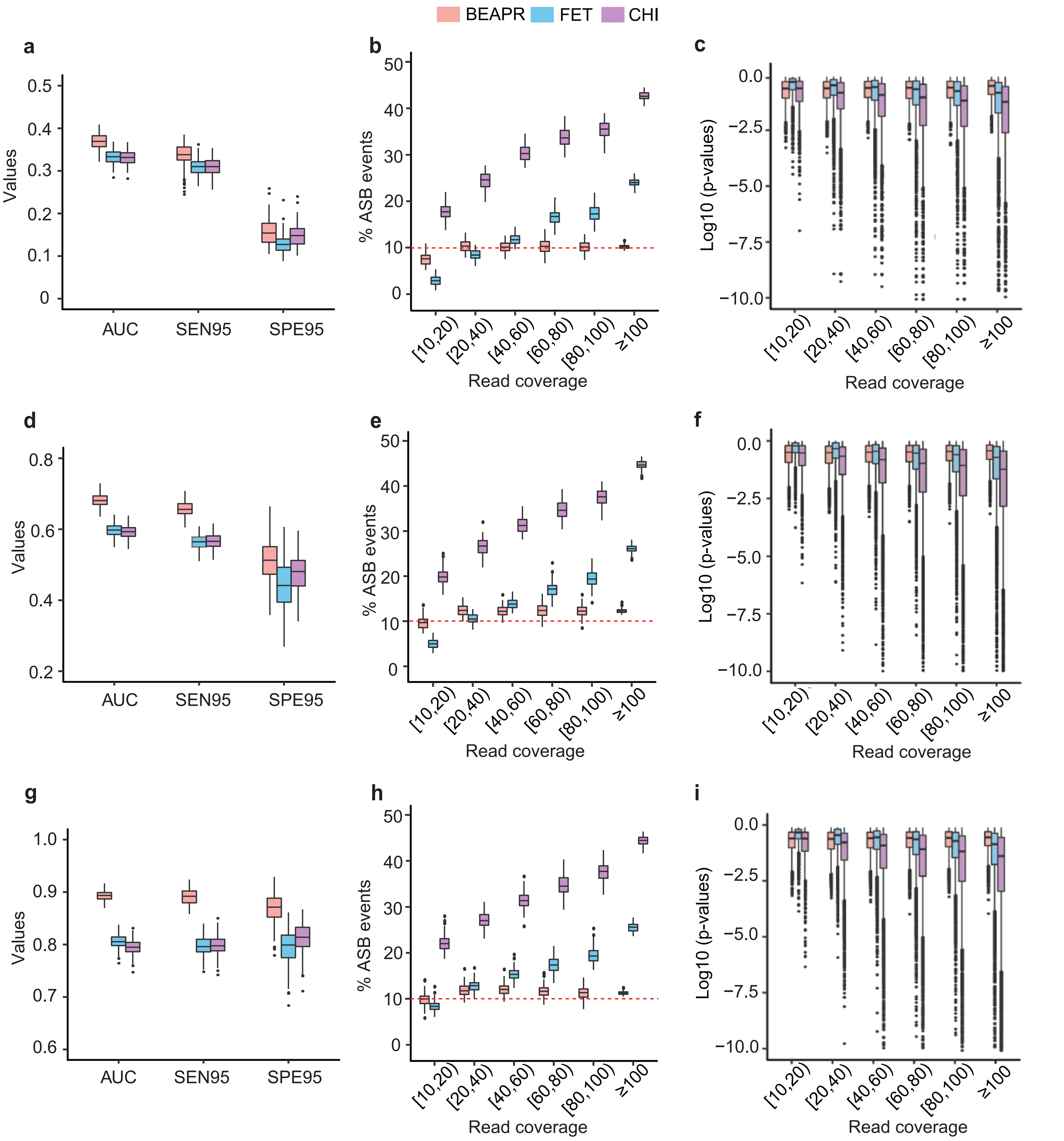

Supplementary Figure 2. Performance comparison on simulated data without crosslinking-induced bias. (a) Performance comparison of 3 methods using simulated data (without simulated crosslinking-induced biases) and true allelic ratio of 0.7 for ASB. Data derived from 1000 simulation experiments each encompassing 5000 SNVs. FET: Fisher' Exact test; CHI: Chi-Squared test; AUC: area under the curve of the precision-recall curve. SEN95: sensitivity at 95% specificity; SPE95: specificity at 95% sensitivity. (b) Percentage of ASB events among all tested SNVs by the 3 methods using simulated data as in (d). The x-axis shows different read coverage bins (using average read coverage of each SNV in two simulated replicates). The red dashed line corresponds to the 10% value, i.e., the percentage of true ASB events in the simulation. (c) Box plots of p values calculated by the 3 methods at different levels of read coverage. (d-f) similar to (a-c), but for true allelic ratio of 0.8 for ASB. (g-i), similar to (a-c), but for true allelic ratio of 0.9 for ASB. Boxplot center lines indicate the median and the boxes extend to lower and upper quartiles with whiskers depicting 1.5 Interquartile range (IQR). The discrete points are the outliers.

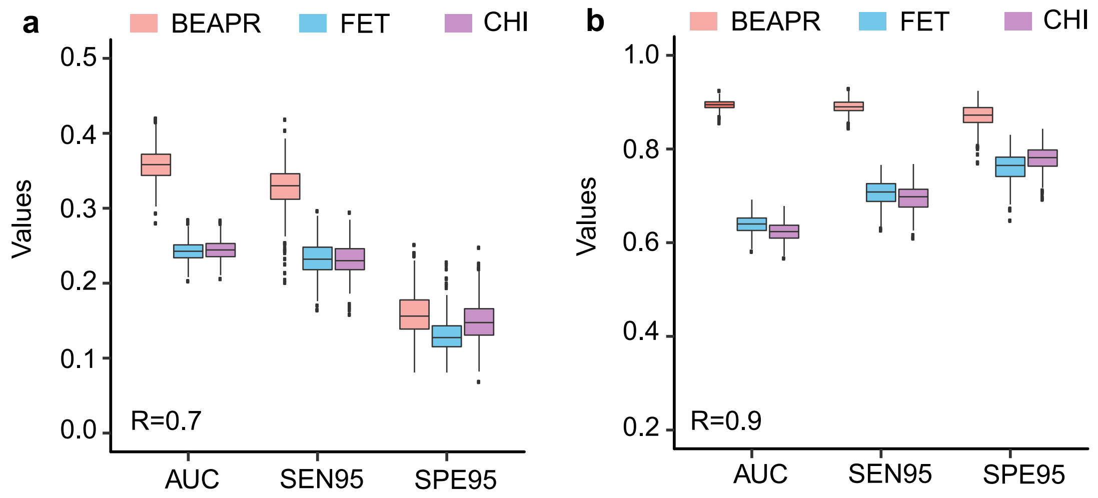

Supplementary Figure 3. Performance comparison of 3 methods using simulated data (with simulated crosslinking-induced biases). Similar as Fig. 1d, but with true allelic ratio of ASB events being (a) 0.7 and (b) 0.9. Boxplot center lines indicate the median and the boxes extend to lower and upper quartiles with whiskers depicting 1.5 IQR. The discrete points are the outliers.

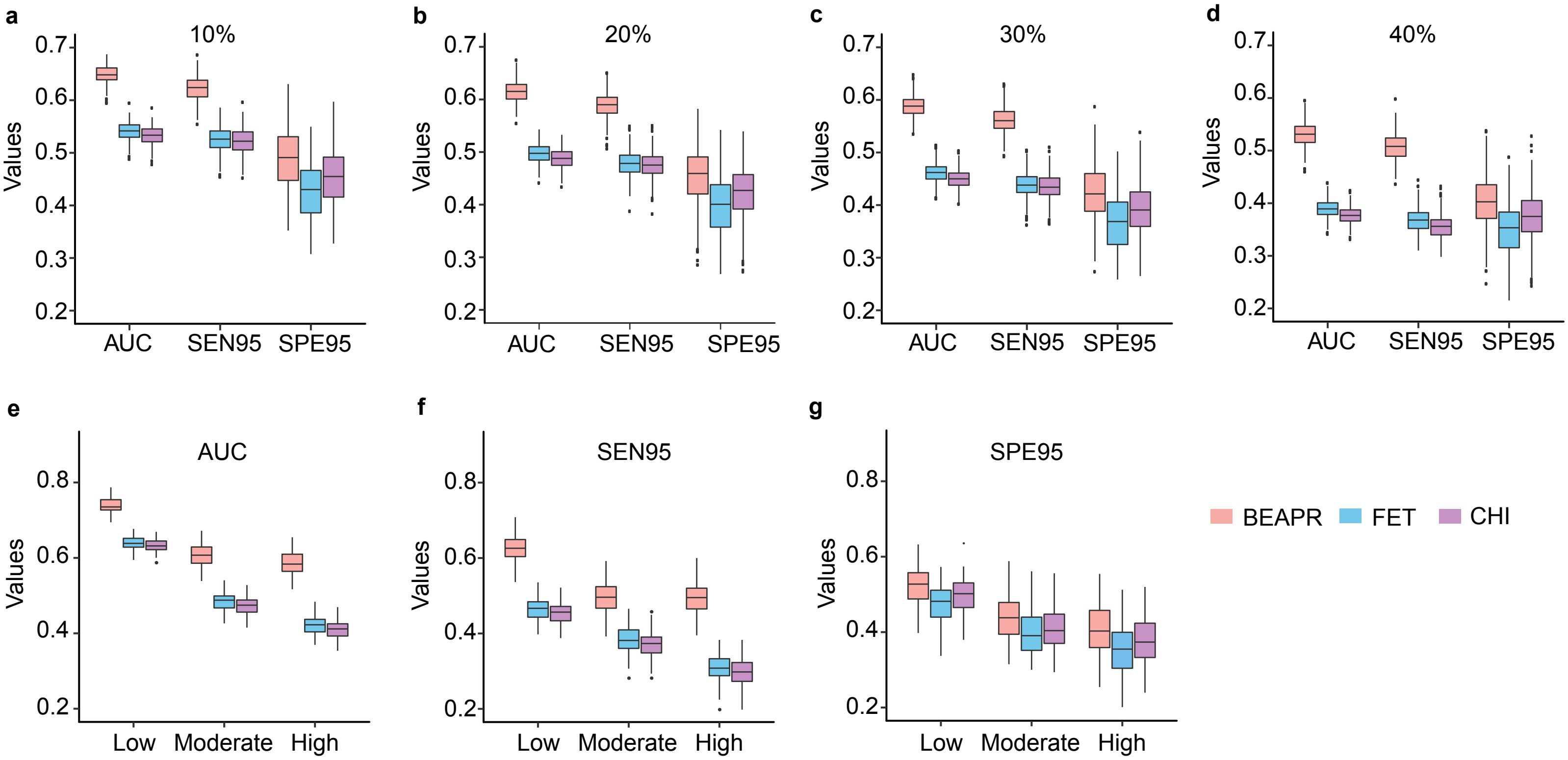

Supplementary Figure 4. Performance comparison of 3 methods using simulated data with outliers (i.e., SNVs with high variances in their read counts, see Methods). (a)-(d) for simulations where 10%, 20%, 30% and 40% of tested SNVs wereoutliers. (e) AUC for 3 subgroups of simulated SNVs in (c) grouped by their standard deviation (std). High: SNVs with allelic read counts whose std was greater than that of 80% of SNVs in the SRSF1 eCLIP data; Moderate: SNVs with allelic read counts whose std was ranked between 70% and 80% of SNVs in the SRSF1 eCLIP data; Low: the rest of SNVs simulated in (c). (f) Similar as (e), but for SEN95 values. (g) Similar as (e), but for SPE95 values. Boxplot center lines indicate the median and the boxes extend to lower and upper quartiles with whiskers depicting 1.5 IQR. The discrete points are the outliers.

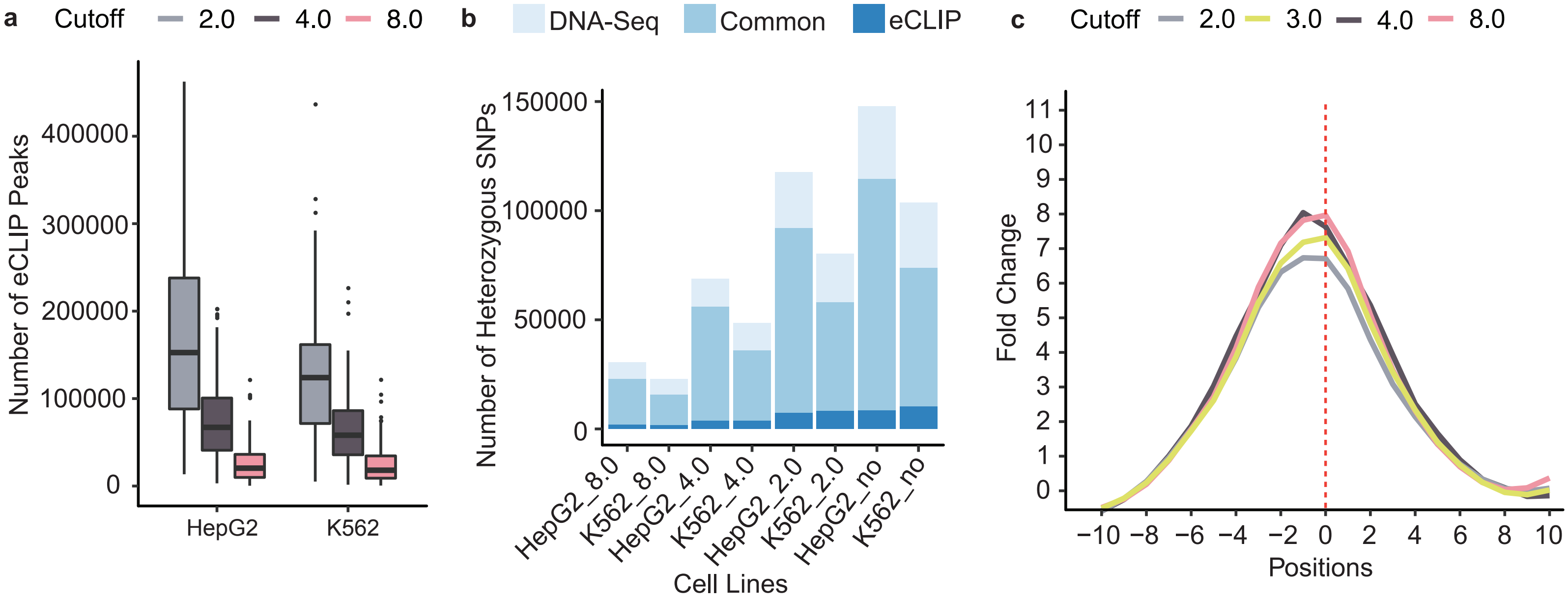

Supplementary Figure 5. Impact of the fold-change cutoff value used to define eCLIP peaks (relative to abundance in SMInput). (a) Number of eCLIP peaks defined using fold-chanage cutoff 2, 4 and 8. Boxplot center lines indicate the median and the boxes extend to lower and upper quartiles with whiskers depicting 1.5 IQR. The discrete points are the outliers. (b) Number of heterozygous SNVs identified via whole-genome DNA sequencing or eCLIP using different cutoffs for eCLIP peak enrichment. HepG2_8.0 shows results using a cutoff value of 8 in HepG2, similarly for others. HepG2_no and K562_no correpond to the results when no fold-change requirement was imposed (thus, the analysis was not restricted to eCLIP peaks). (c) Similar to Fig. 3a-f, the motif enrichment of RBFOX2 in HepG2 cells. Similar levels of motif enrichment were observed given the cutoff 4 and 8. This enrichment was reduced when 2 or 3 was used as the cutoff.

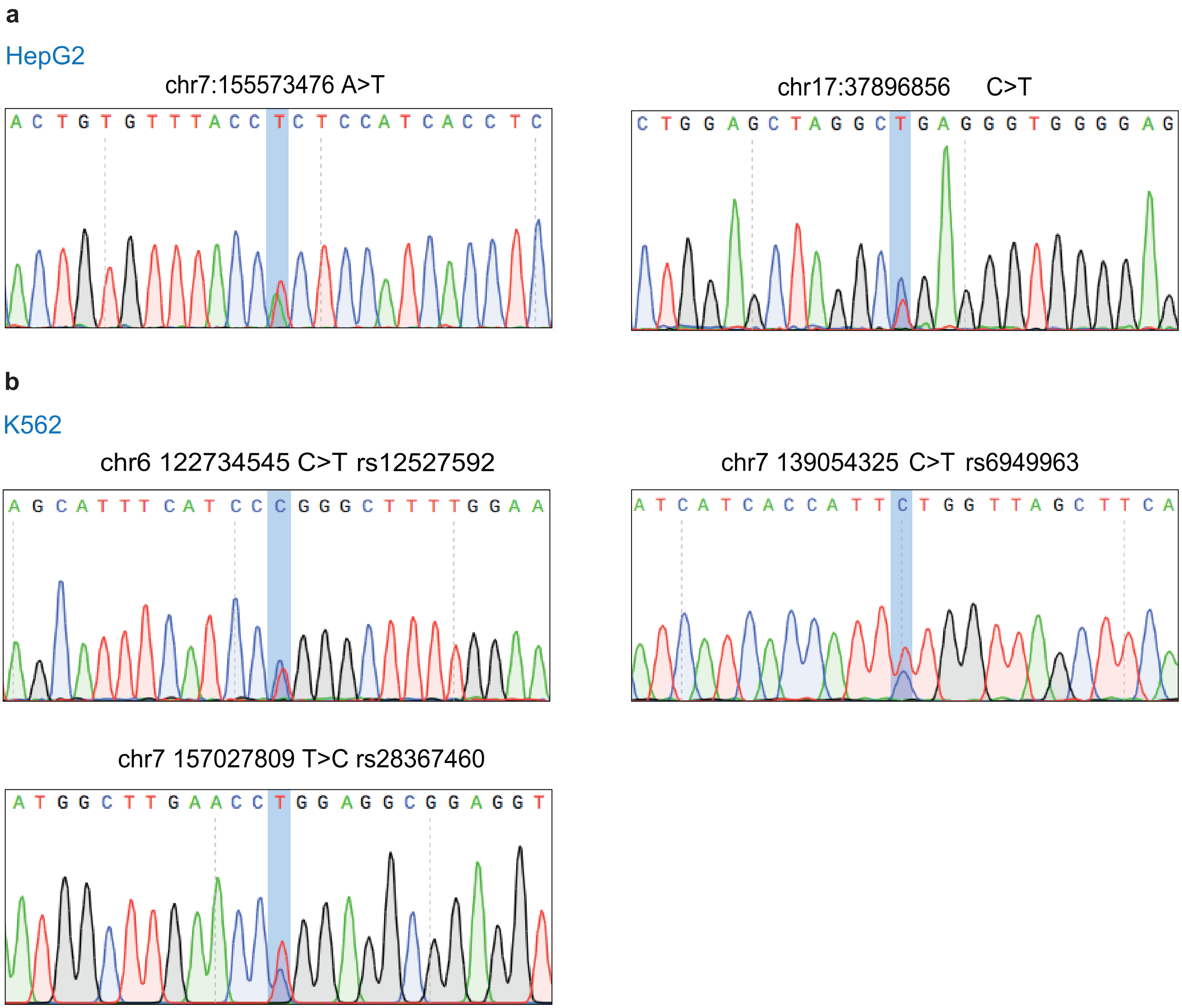

Supplementary Figure 6. Sanger sequencing of DNA to confirm BEAPR-predicted heterozygous SNPs. (a) HepG2, (b) K562

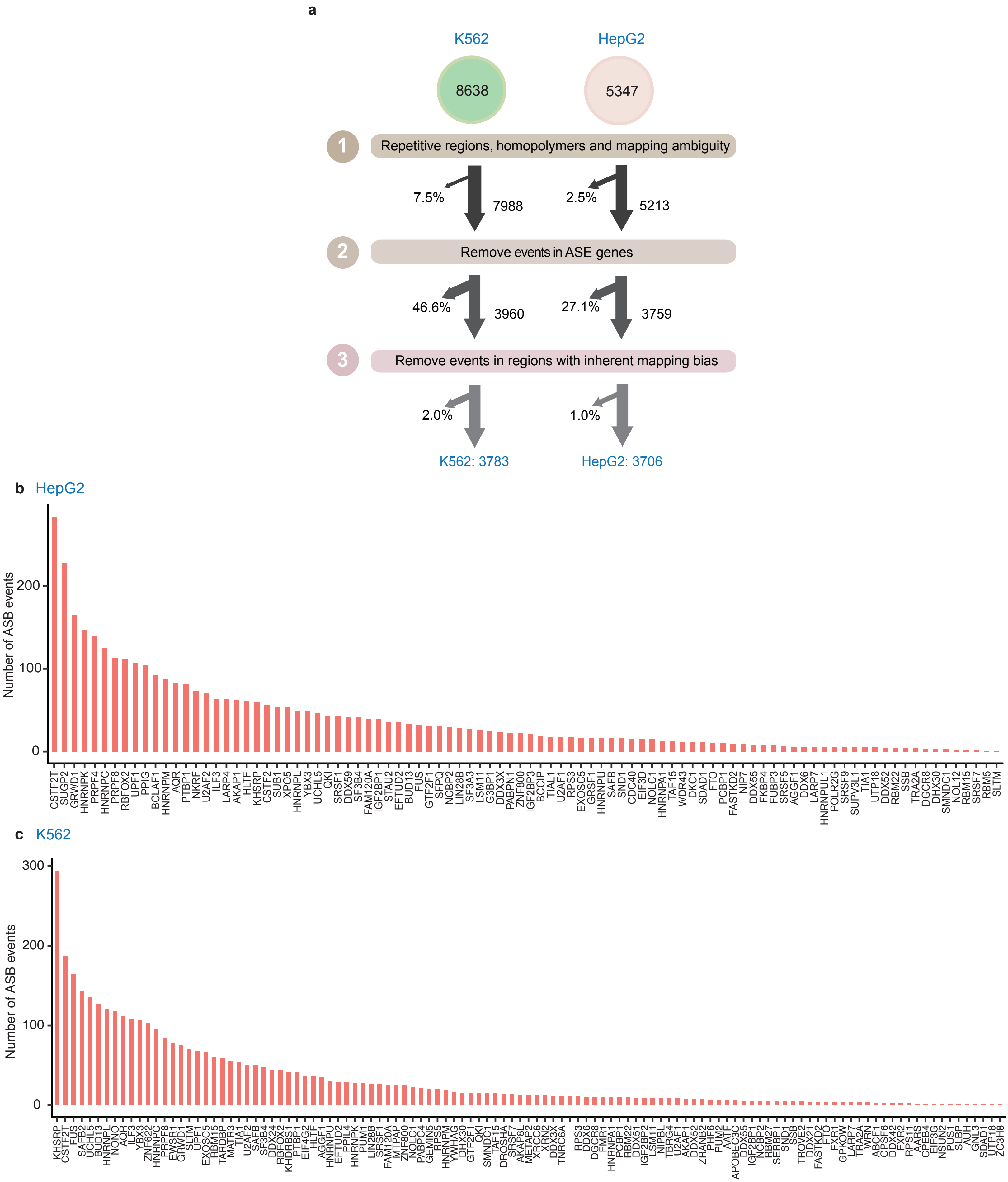

Supplementary Figure 7. ASB events identified in ENCODE eCLIP data. (a) Number and percentage of ASB events (union of all RBPs) filtered in each step of post-processing (Methods). (b) Number of final ASB events for each RBP in HepG2 cells. (c) Similar as (b), K562 cells.

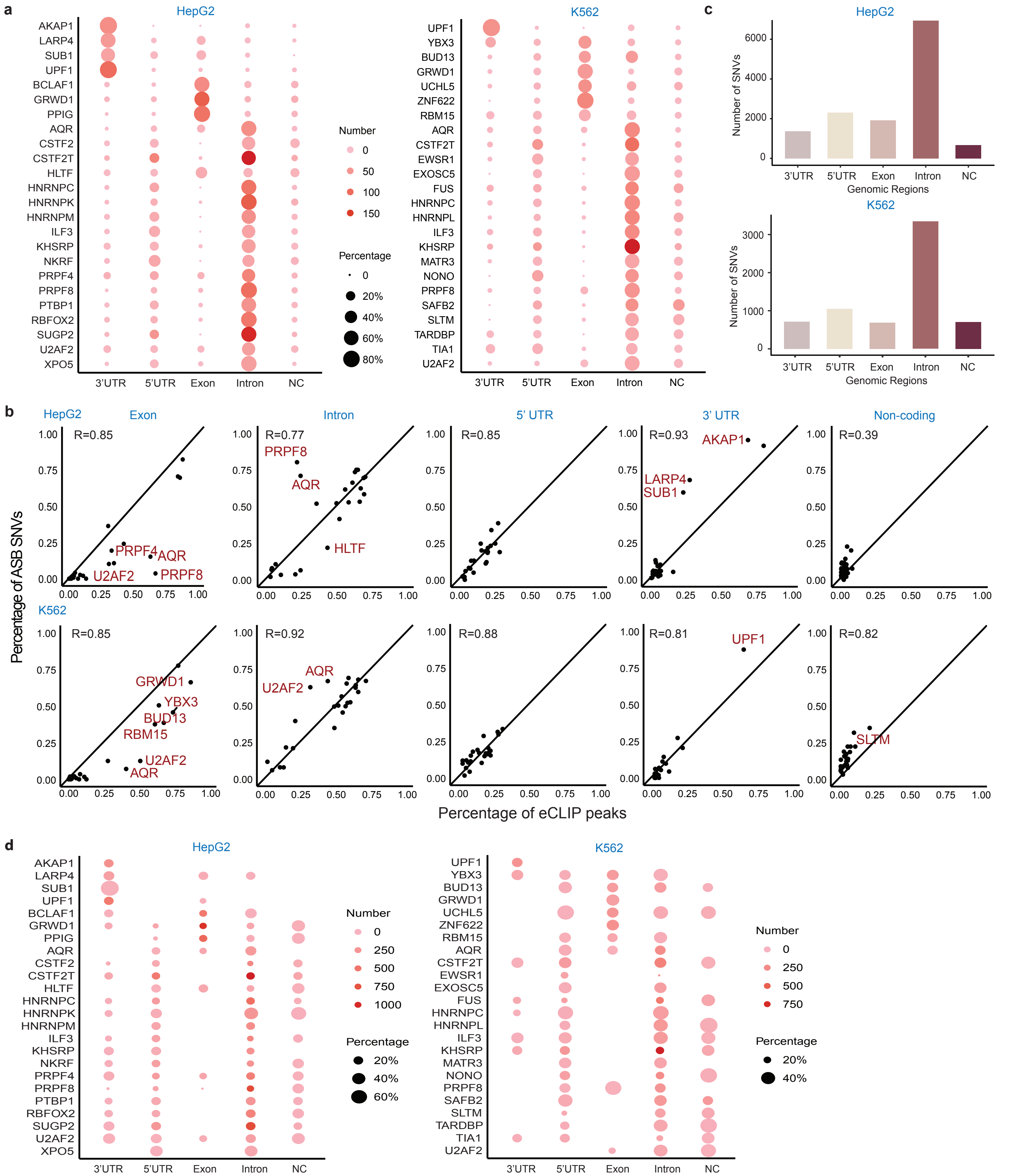

Supplementary Figure 8. ASB events in different types of genomic regions. (a) Distribution of ASB SNVs in different types of genomic regions. Only RBPs with ≥50 ASB events are shown. The category "Exon" refer to coding exons. NC: non-coding transcripts. The color of the dots denotes number of events. The size of the dots corresponds to the percentage of SNVs in each region for each RBP. (b) Correlation between the percentage of ASB SNVs and that of eCLIP peaks in each type of genomic region. R: pearson correlation coefficient. (c) Distributions of testable heterozygous SNVs across genomic regions. (d) Distributions of testable hterozygous SNVs in different types of genomic regions for each RBP. Only RBPs with ≥50 ASB events are shown. Data points with <20 testable SNVs were disgarded. The color of the dots denotes number of testable heterozygous SNVs. The size of the dots corresponds to the percentage of predicted ASB SNVs among the testable SNVs in each region for each RBP.

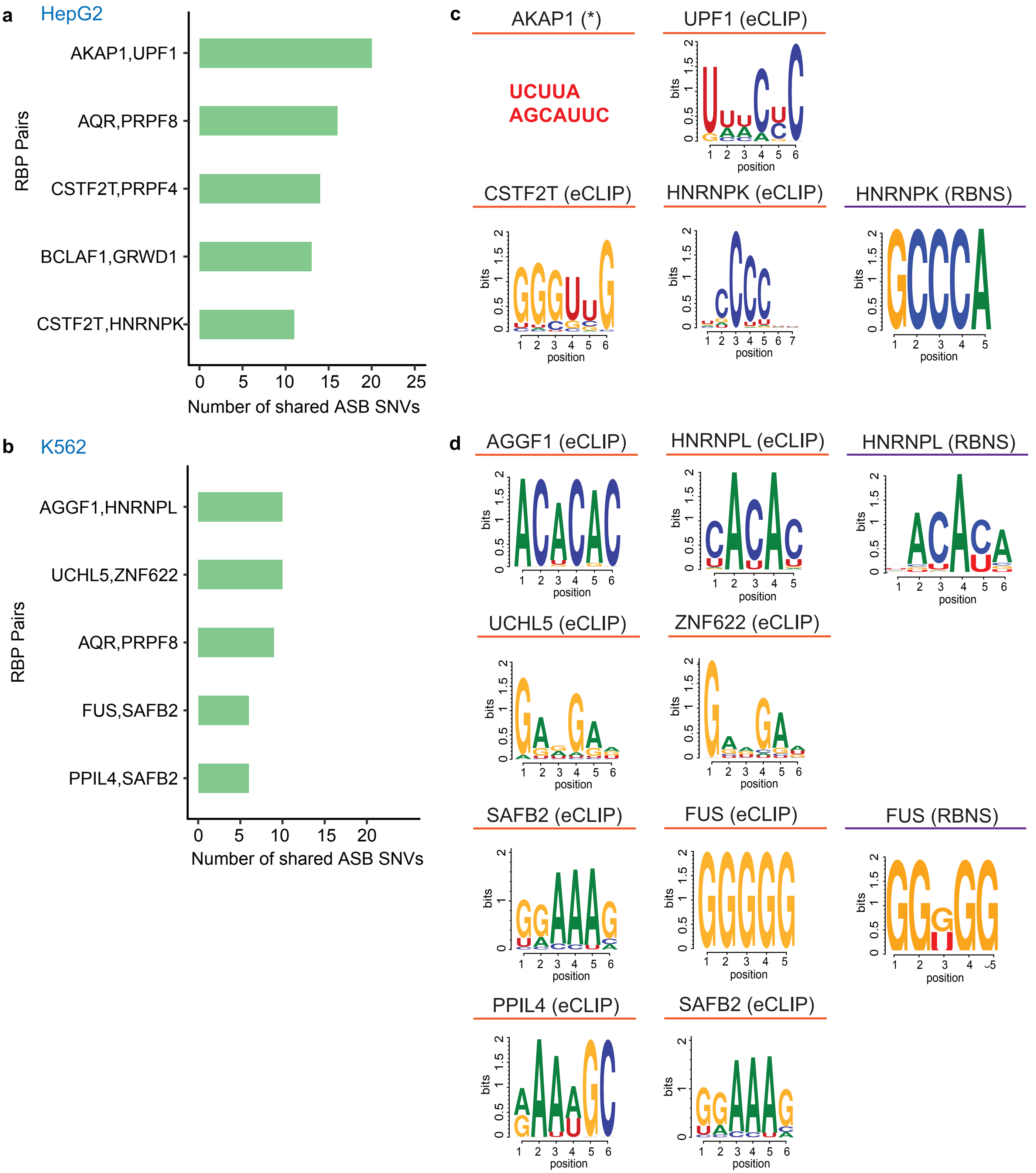

Supplementary Figure 9. ASB events common to pairs of RBPs. (a) The number of shared ASB SNVs by each pair of RBPs in HepG2 cells. Only the top 5 pairs of RBPs with the most shared events are shown. (b) Similar as (a), for K562 cells. (c) Motifs of RBPs in (a) derived from eCLIP or RBNS data (Van Nostrand et al, doi: https://doi.org/10.1101/179648). *The binding sequences of AKAP1 were obtained from the RBPDB database (Cook et.al., PMID:21036867). Some proteins have no known motifs. (d) Similar as (c), for RBPs in (b). Some RBP pairs, such as AGGF1-HNRNPL, UCHL5-ZNF662 and PPIL4-SAFB2, share similar motifs, consistent with existence of shared ASB events. However, other RBP pairs do not have known motifs that are similar. These proteins may interact with each other and bind to RNA in complexes.

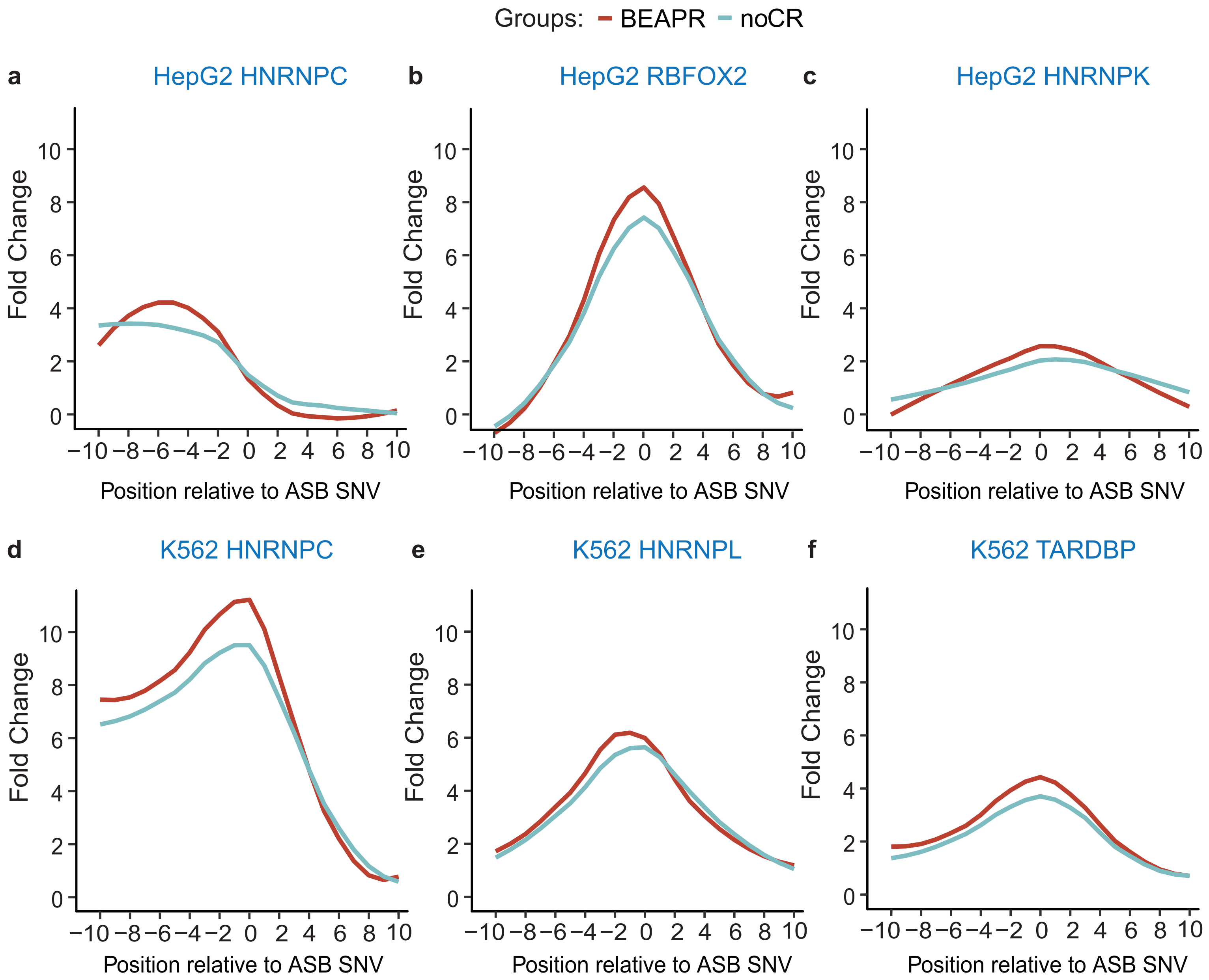

Supplementary Figure 10. Correction for crosslinking-induced bias in BEAPR improves ASB prediction. Similar to Fig 3, motif enrichment is used as a validation of the predicted ASB events.BEAPR: full version of BEAPR with correction for crosslinking-induced bias. noCR: BEAPR without bias correction.

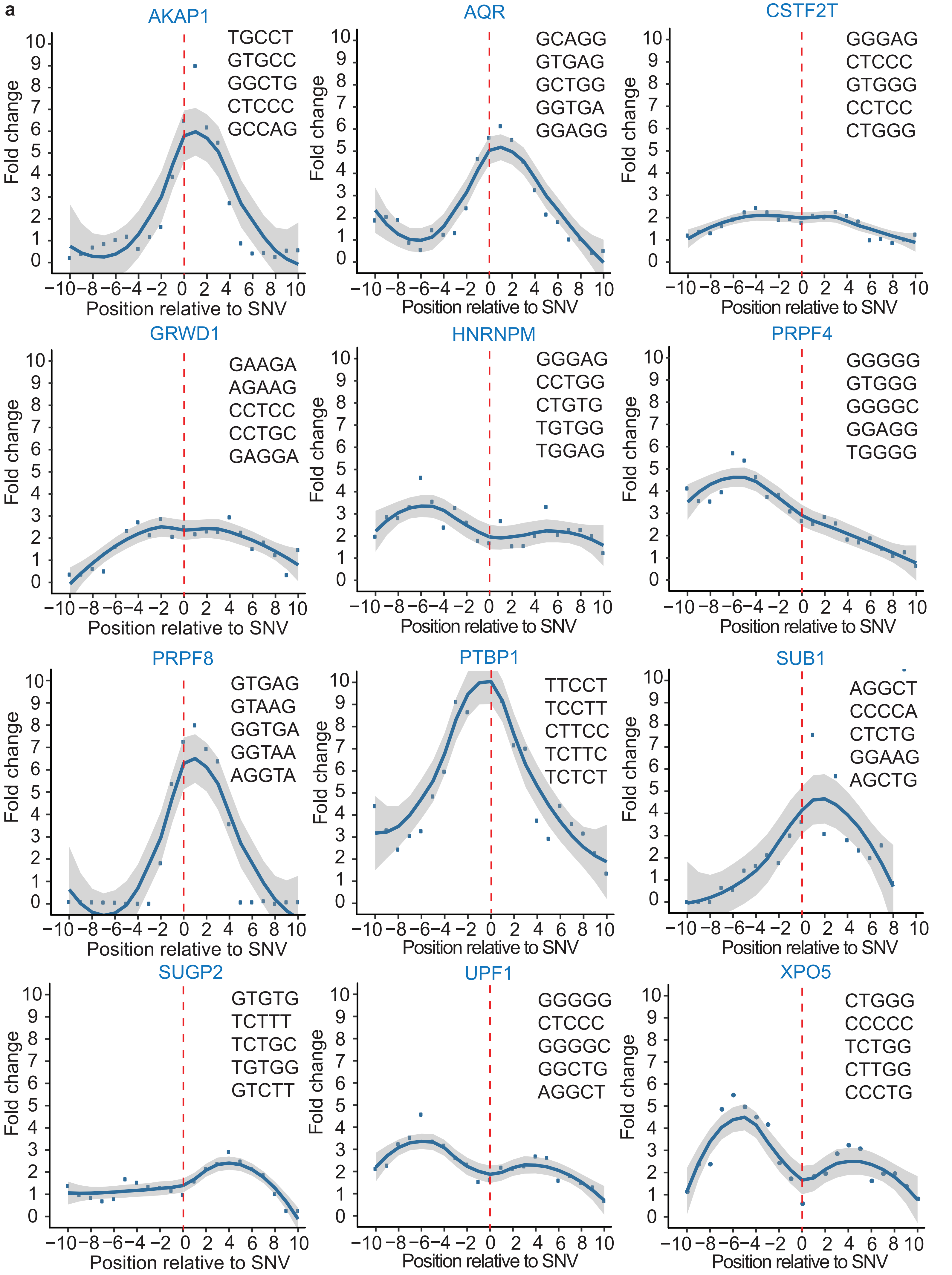

Supplementary Figure 11a. Positional enrichment of pentamers around ASB SNV sites (x = 0) in HepG2 cells. The top 5 pentamers that are most enriched in regions of ASB were identified, and listed in each panel. Similar to Fig 3 a-f, the regression curves (blue lines) and 95% confidence intervals (shaded areas) of the average fold change shown in the panels.

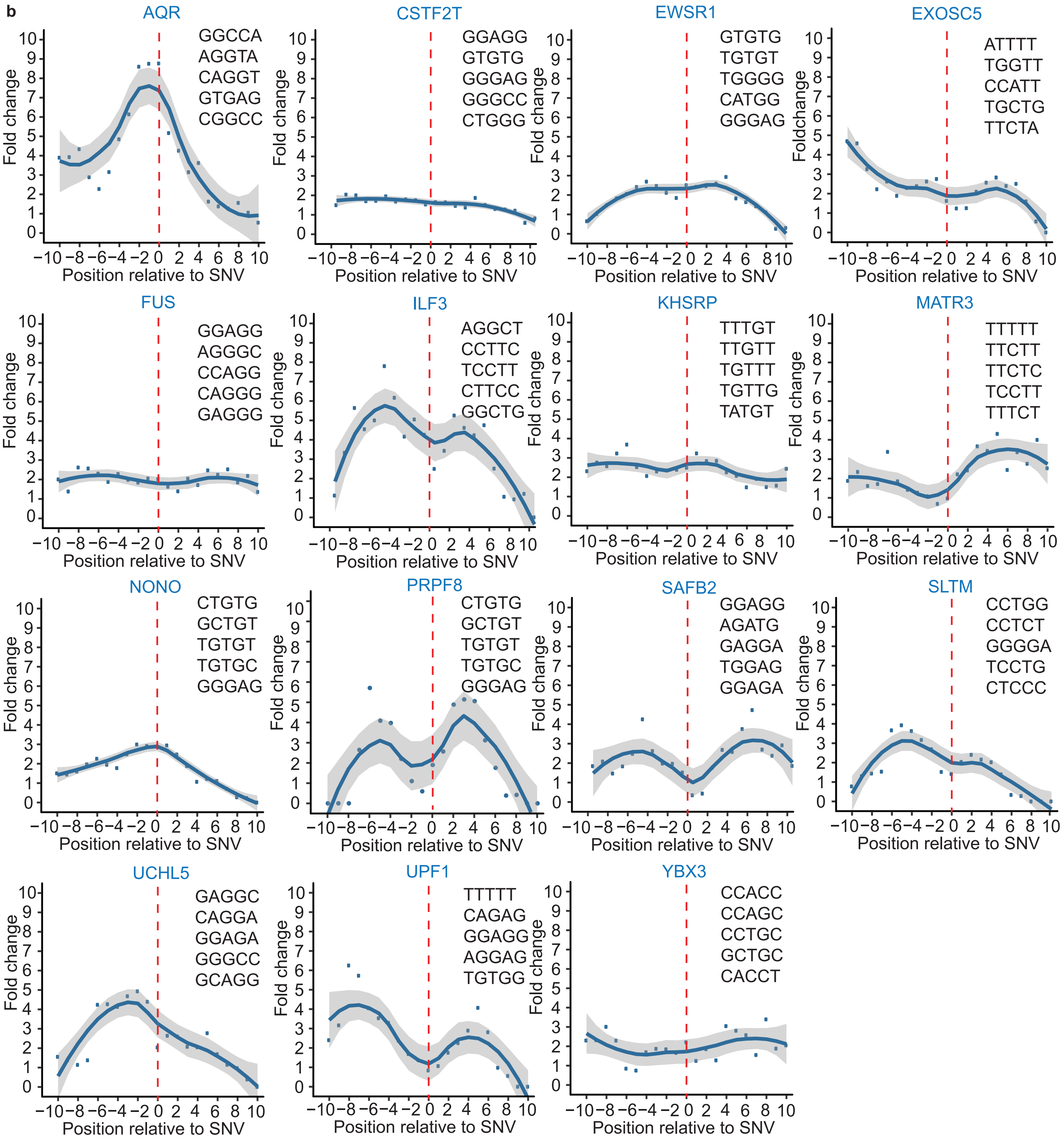

Supplementary Figure 11b. Positional enrichment of pentamers around ASB SNV sites (x = 0) in K562 cells, similar as S11a. Similar to Fig 3 a-f, the regression curves (blue lines) and 95% confidence intervals (shaded areas) of the average fold change shown in the panels.

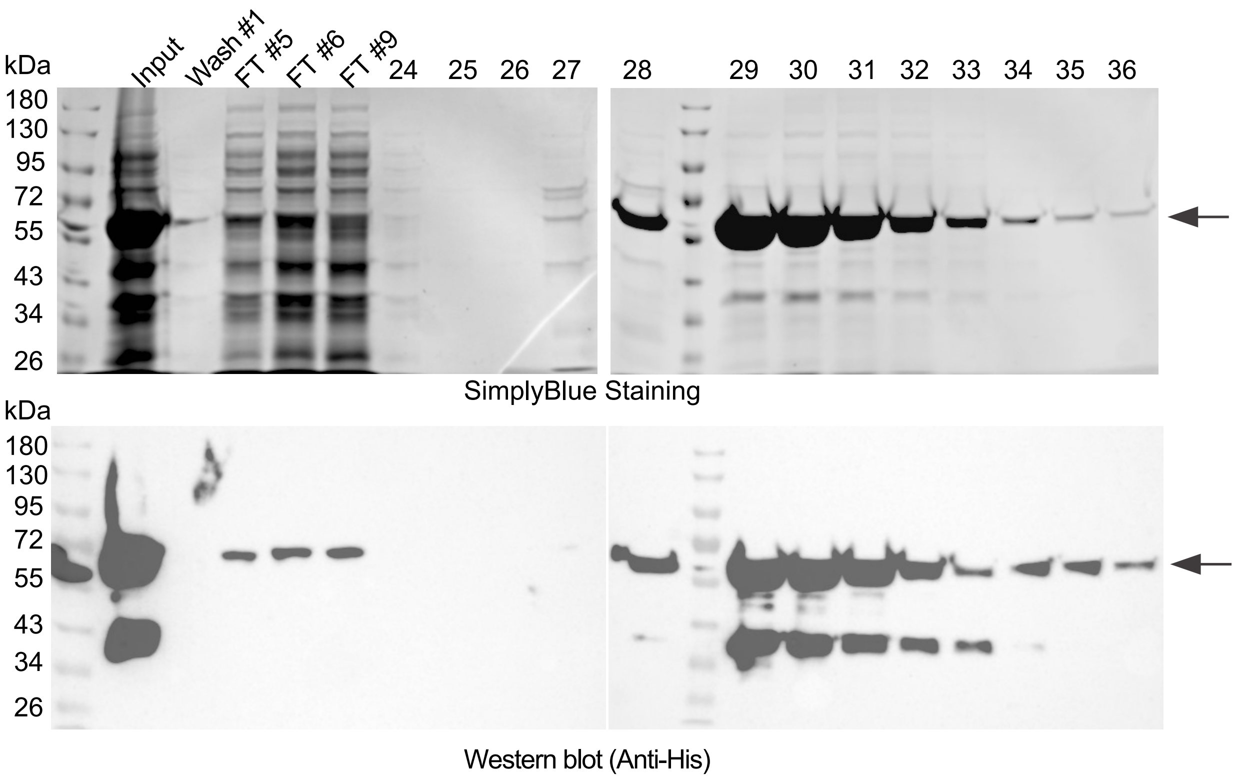

Supplementary Figure 12. Recombinant PTBP1 bacterial overexpression and electrophoretic mobilty shift assay (EMSA). Baterial overexpression of human PTBP1. PTBP1-pET28a plasmid was obtained from Dr. Doug Black's lab and PTBP1 recombinant proteins were purified from BL21 Star (DE3) using the HisTrap purification column. Detailed purification conditions are described in Methods. Fractions from each purification step were loaded onto 8% SDS-PAGE. (FT: flow through, elution 24-36). Purified PTBP1 proteins were confirmed by SimplyBlue Safe staining (top) and western blot (bottom). Extra bands of smaller protein sizes are likely due to degradation. 28 to 33 fractions were used, followed by 20K dialysis. (bottom). Extra bands of smaller protein sizes are likely due to degradation.

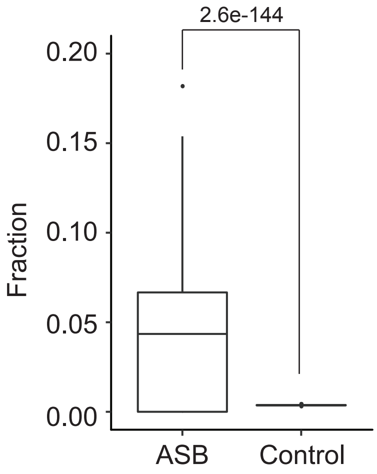

Supplementary Figure 13. Similar as Fig. 4d, fraction of ASB events located in sQTL exons or within 500nt in their flanking introns among the union of ASB events of all splicing factors in the HepG2 or K562 data. sQTL data were extracted from the TCGA project for Liver hepatocellular carcinoma. Boxplot center lines indicate the median and the boxes extend to lower and upper quartiles with whiskers depicting 1.5 IQR. The discrete points are the outliers.P value was calculated by Steduent's *t*-test.

a

RBP with ASB events in 3’ UTR regions

	RBPs	ASB SNVs in 3’ UTR
HepG2	UPF1	92
	AKAP1	57
	LARP4	41
K562	UPF1	56
	YBX3	28
	KHSRP	17

b

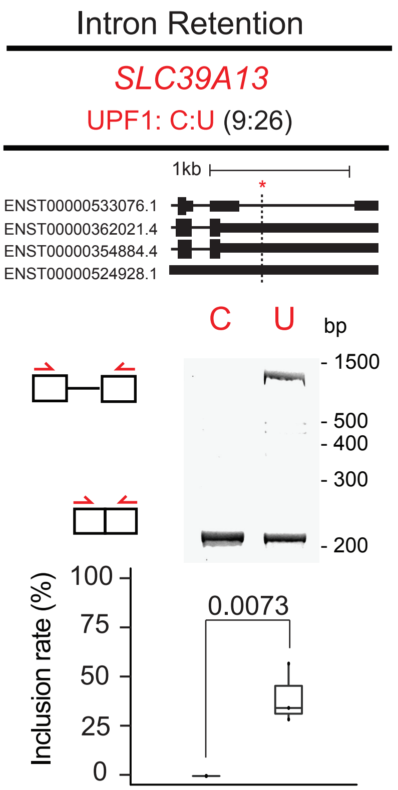

Supplementary Figure 14. ASB events in 3’ UTRs. (a) The top 3 RBPs in each cell line with the highest number of ASB SNVs in 3’ UTRs. (b) Experimental validation of a SNP in the *SLC39A13* gene for its function in influencing intron retention, similar as Fig. 4d. Boxplot center lines indicate the median and the boxes extend to lower and upper quartiles with whiskers depicting 1.5 IQR. P value was calculated by Steduent’s *t*-test. (Images are cropped, with uncropped images in Supplementary Figure 16.)

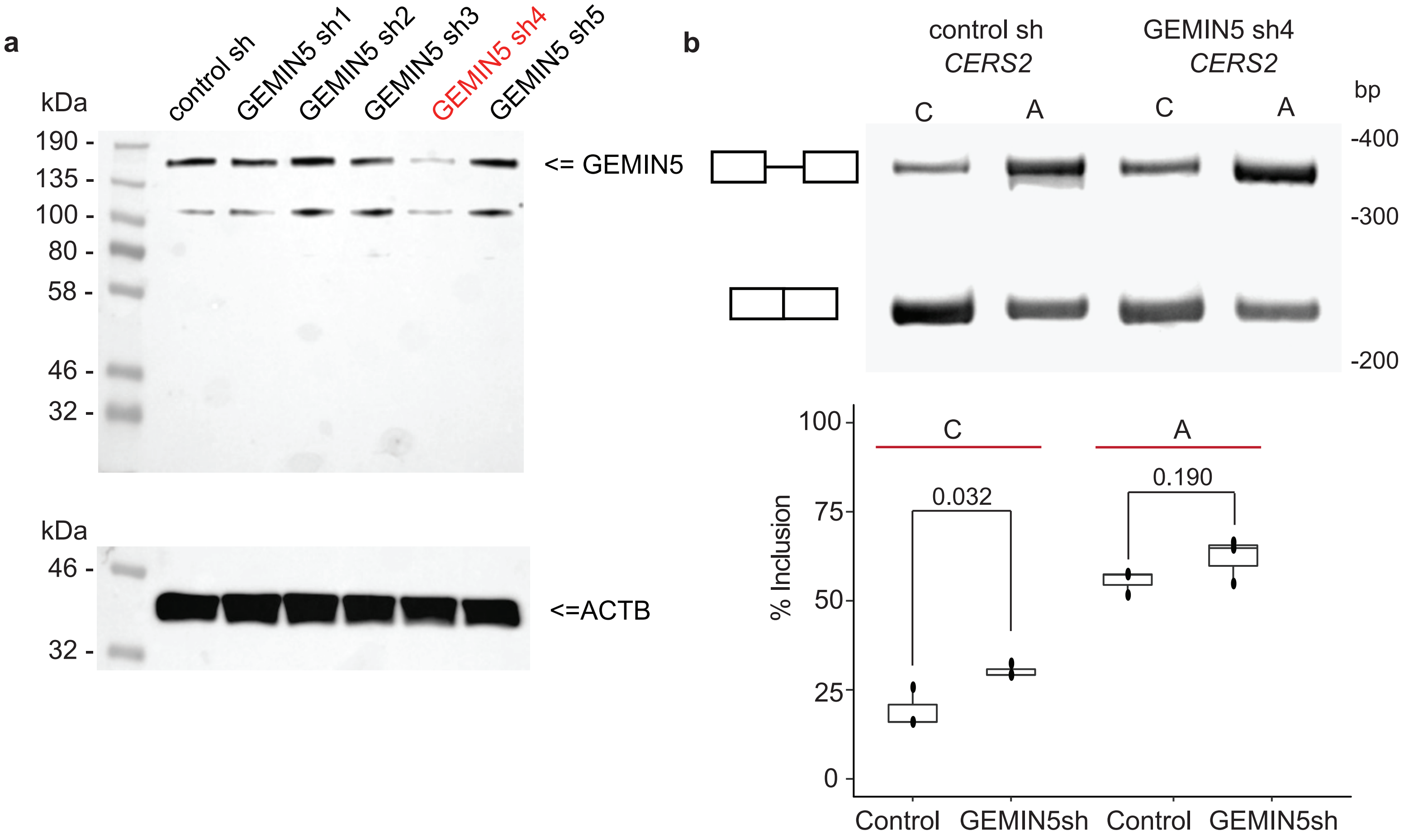

Supplementary Figure 15. Impact of GEMIN5 knockdown (KD) on splicing of the minigene of *CERS2*. (a) Western blot of GEMIN5 protein expression in HeLa cells transfected with control shRNA (sh) or one of the five (sh1-5) GEMIN5-targeting shRNAs. β-Actin was used as sample loading control. GEMIN5 sh4 has the strongest KD effect, which was used in (b). (b) Similar to Fig. 6c, splicing of reporter genes containing one of the two alternative alleles in the *CERS2* gene in the control and KD cells. (Images are cropped, with uncropped images in Supplementary Figure 16.) P values were calculated by Student's *t*-test. Boxplot center lines indicate the median and the boxes extend to lower and upper quartiles with whiskers depicting 1.5 IQR.

Supplementary Figure16a. Uncropped images of Fig.3g

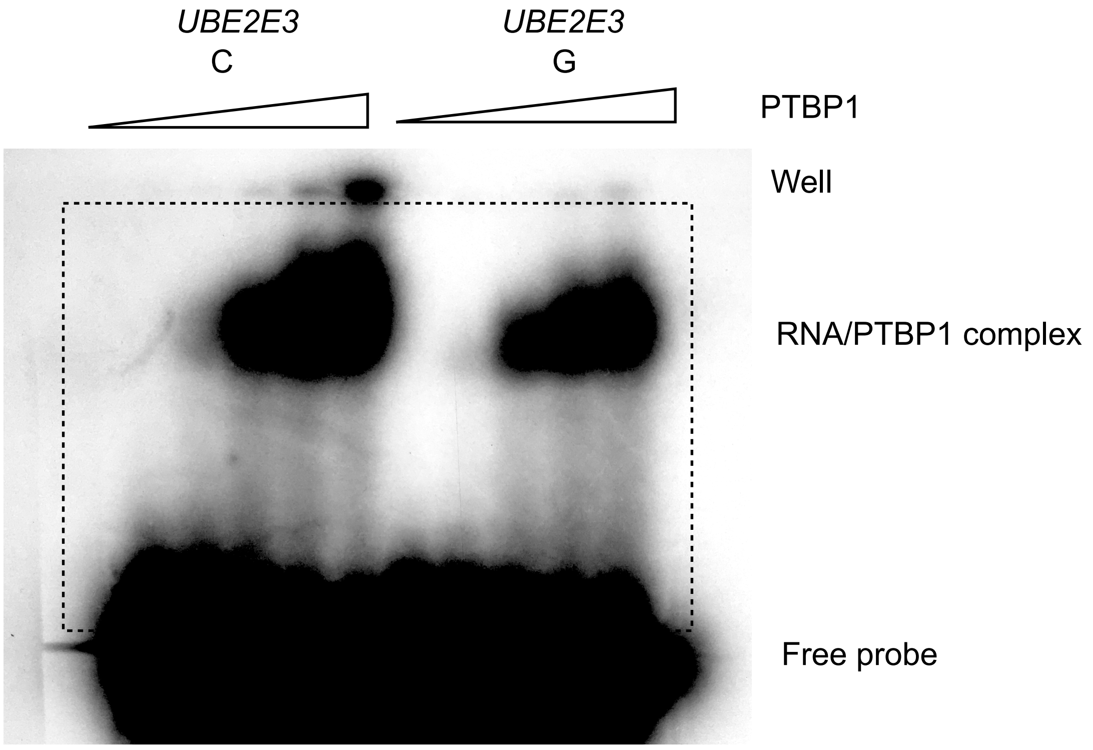

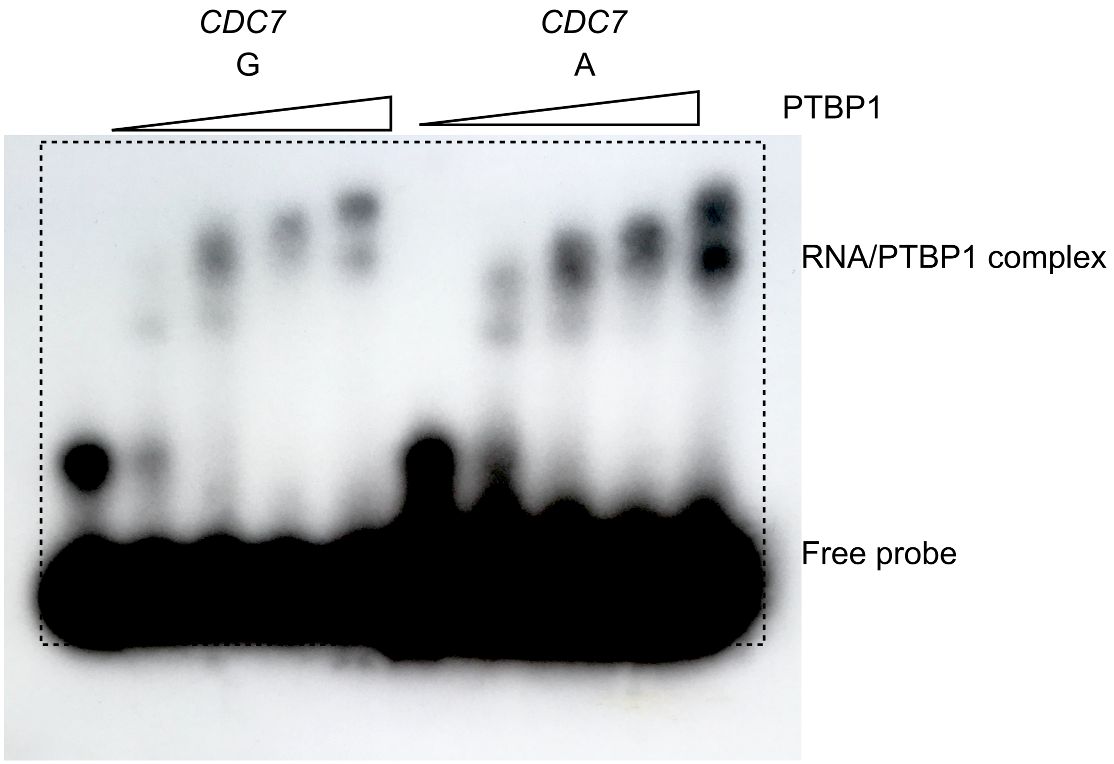

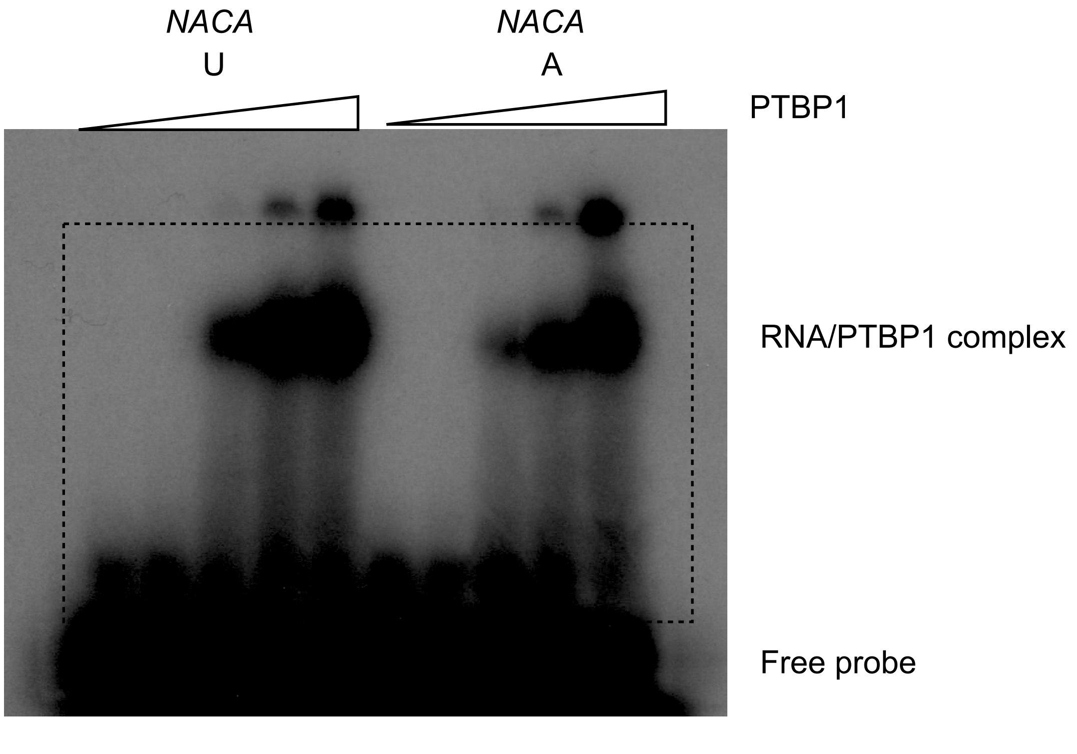

Supplementary Figure 16b. Uncropped images of Fig.4e (exon skipping events)

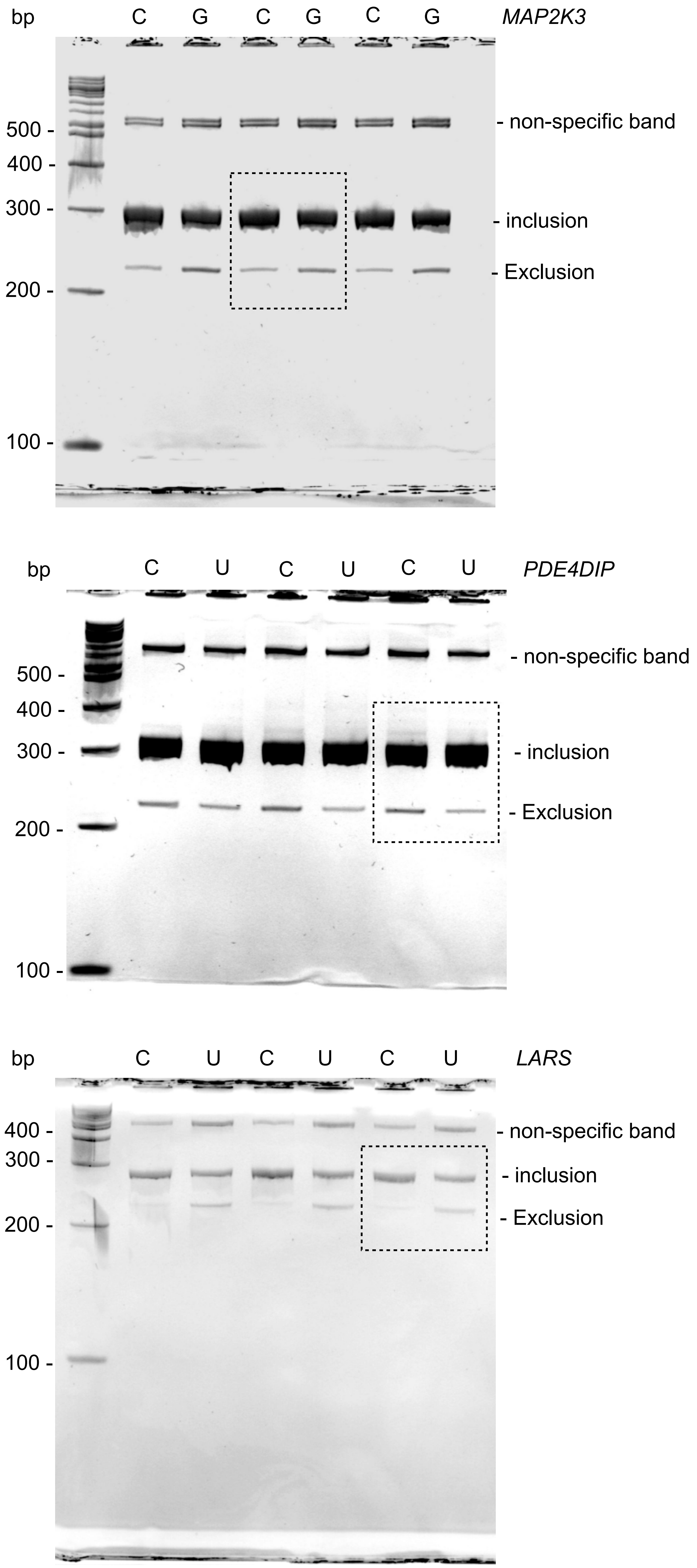

Supplementary Figure 16c. Uncropped images of Fig.4e (Intron retention events)

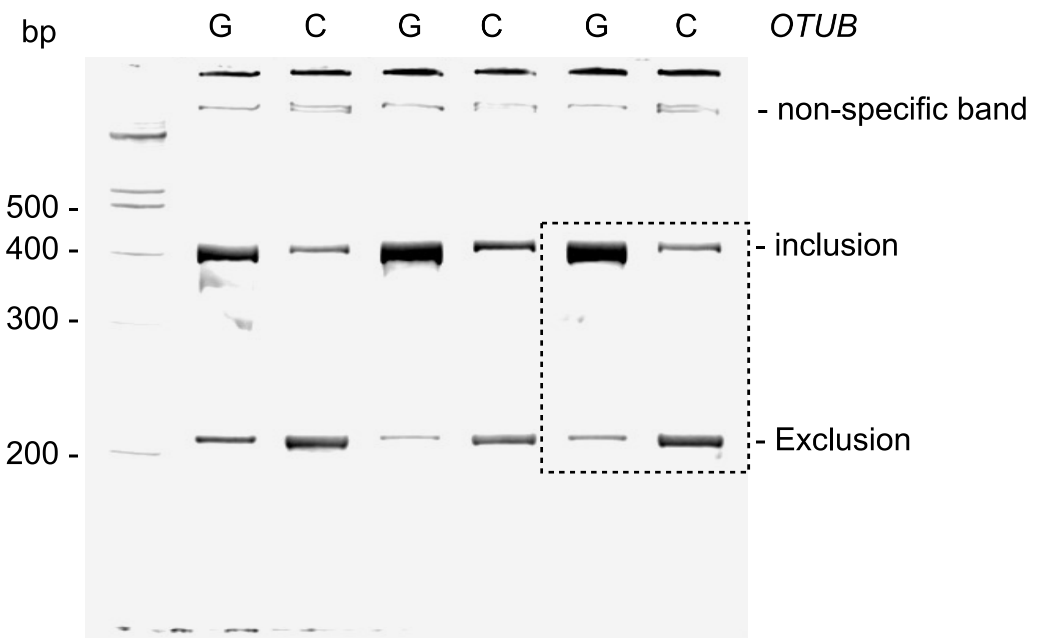

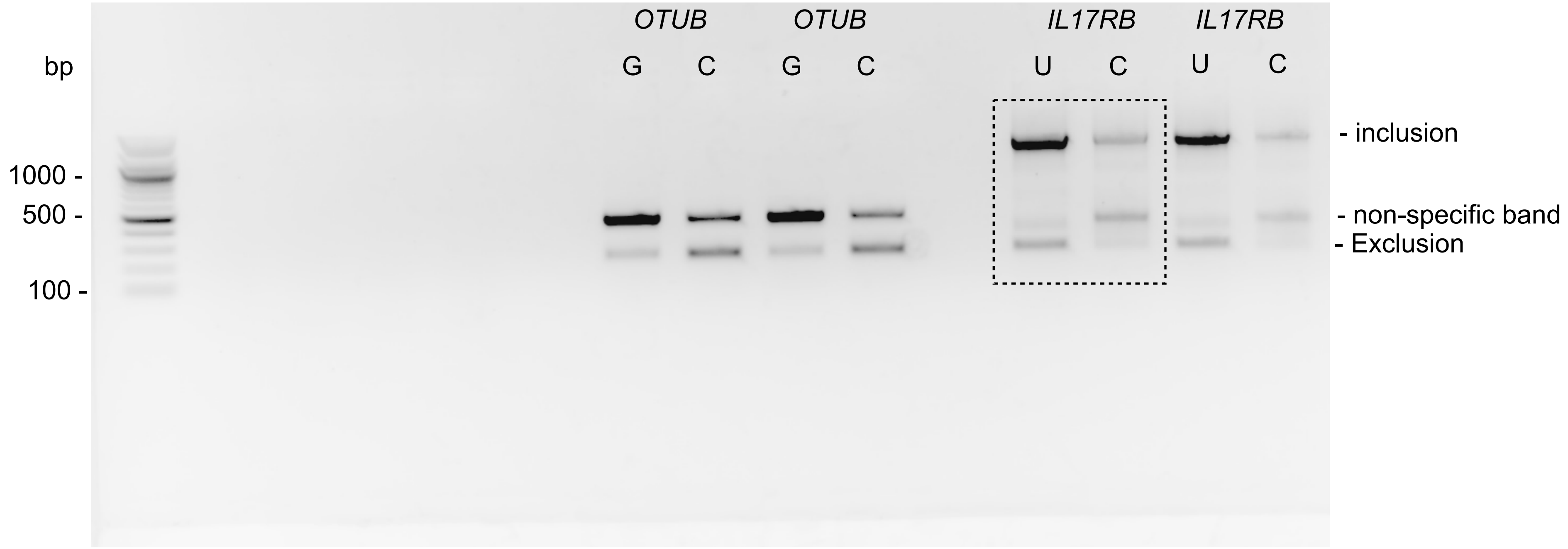

Supplementary Figure 16d. Uncropped images of Fig.6c.

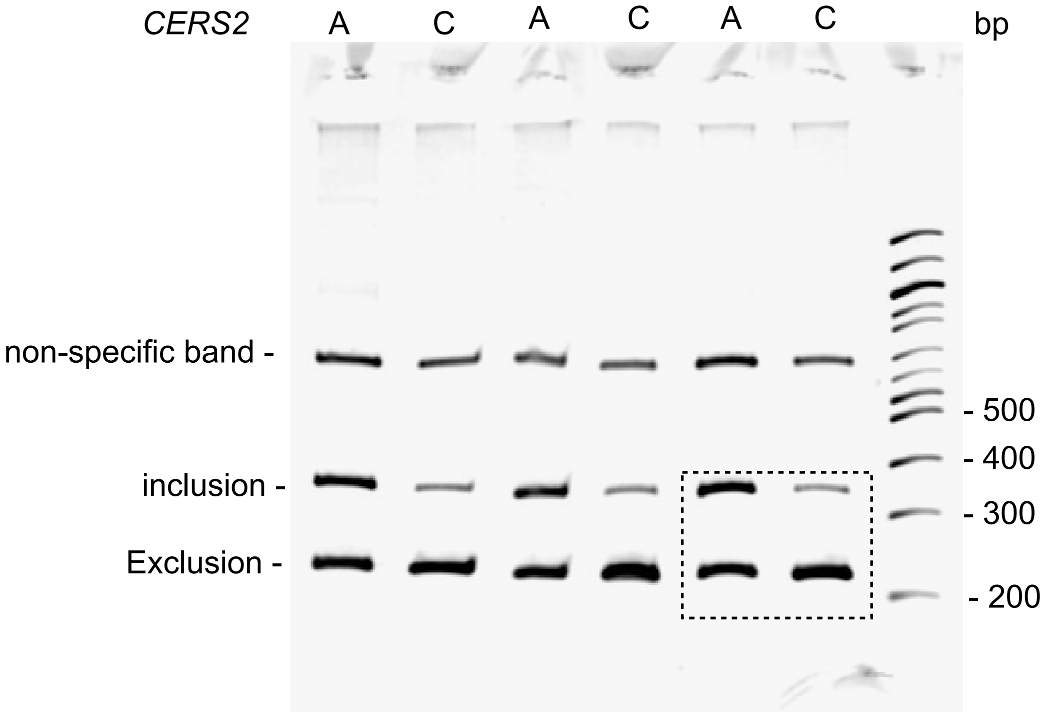

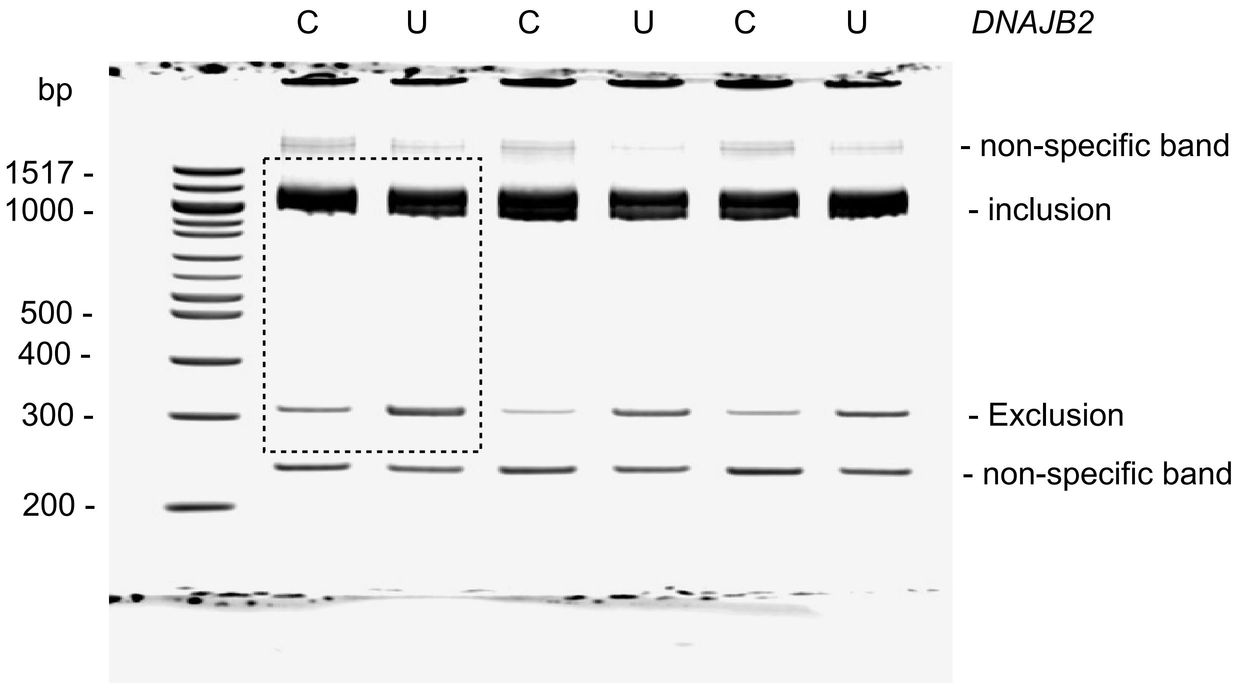

Supplementary Figure 16e. Uncropped images of Fig.S14b.

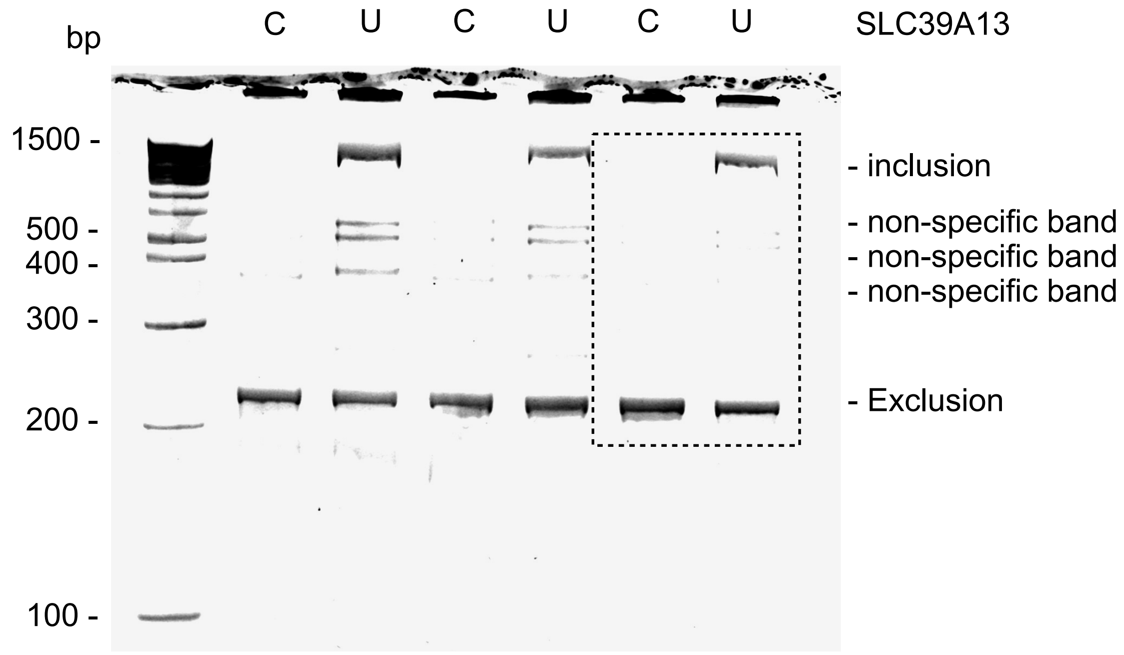

Supplementary Figure 16f. Uncropped images of Fig.S15b.

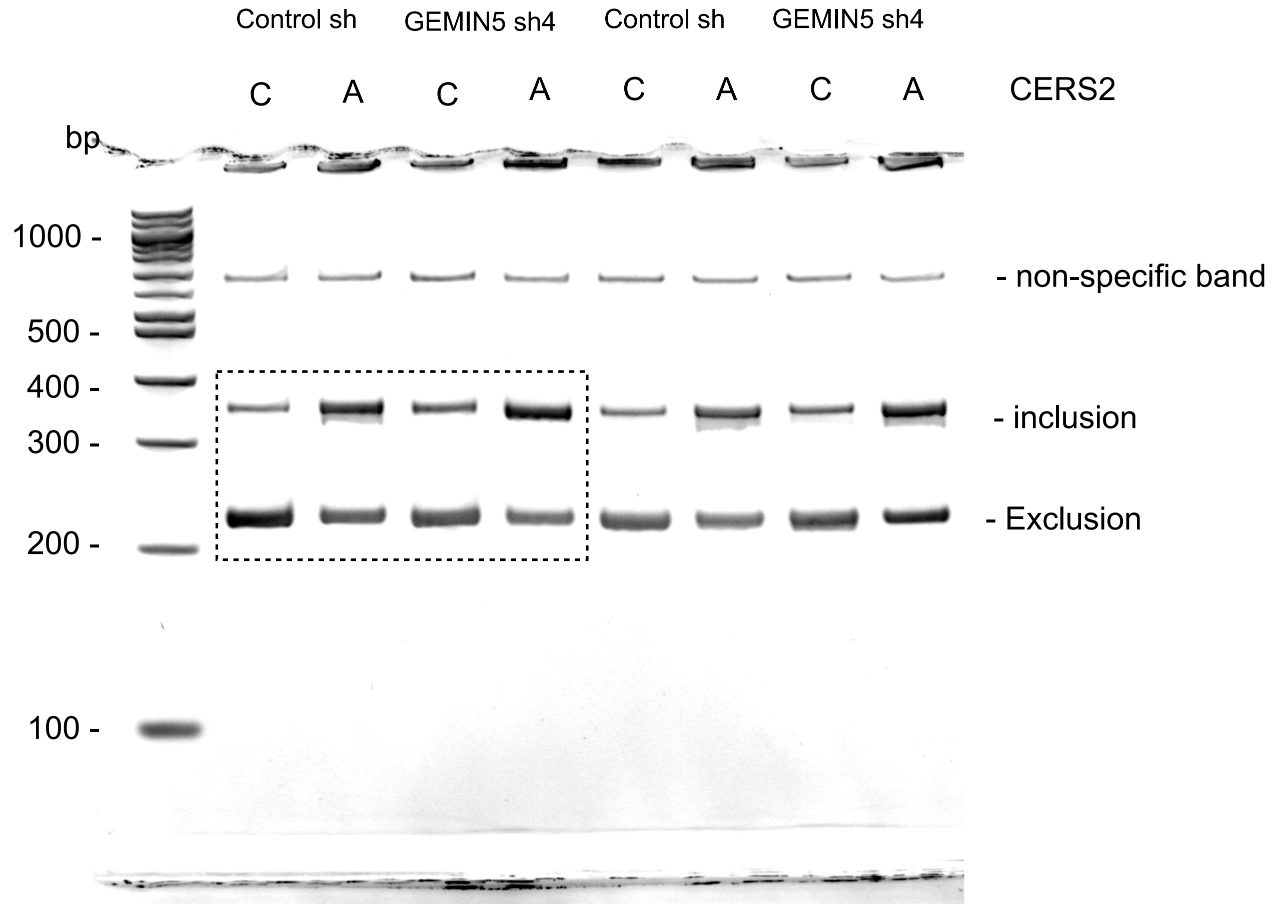

Supplement: Supplementary file 1 — Supplementary Information [file 41467_2019_9292_MOESM1_ESM.pdf]
