# Supplementary material for: Allele-specific binding of RNA-binding proteins reveals functional genetic variants in the RNA
Source: Nat Commun. 2019 Mar 22;10:1338. doi: 10.1038/s41467-019-09292-w (PMC6430814; doi:10.1038/s41467-019-09292-w)
Supplement: Supplementary file 2 — Reporting Summary [file 41467_2019_9292_MOESM2_ESM.pdf]

nature research

Corresponding author(s): Xinshu Xiao

Last updated by author(s): 2019/02/23

Reporting Summary

Nature Research wishes to improve the reproducibility of the work that we publish. This form provides structure for consistency and transparency in reporting. For further information on Nature Research policies, see Authors & Referees and the Editorial Policy Checklist.

Statistics

For all statistical analyses, confirm that the following items are present in the figure legend, table legend, main text, or Methods section.

n/a	Confirmed	
☐	☒	The exact sample size (*n*) for each experimental group/condition, given as a discrete number and unit of measurement
☐	☒	A statement on whether measurements were taken from distinct samples or whether the same sample was measured repeatedly
☐	☒	The statistical test(s) used AND whether they are one- or two-sided<br>*Only common tests should be described solely by name; describe more complex techniques in the Methods section.*
☒	☐	A description of all covariates tested
☐	☒	A description of any assumptions or corrections, such as tests of normality and adjustment for multiple comparisons
☐	☒	A full description of the statistical parameters including central tendency (e.g. means) or other basic estimates (e.g. regression coefficient) AND variation (e.g. standard deviation) or associated estimates of uncertainty (e.g. confidence intervals)
☐	☒	For null hypothesis testing, the test statistic (e.g. *F*, *t*, *r*) with confidence intervals, effect sizes, degrees of freedom and *P* value noted<br>*Give P values as exact values whenever suitable.*
☒	☐	For Bayesian analysis, information on the choice of priors and Markov chain Monte Carlo settings
☒	☐	For hierarchical and complex designs, identification of the appropriate level for tests and full reporting of outcomes
☐	☒	Estimates of effect sizes (e.g. Cohen's *d*, Pearson's *r*), indicating how they were calculated

Our web collection on statistics for biologists contains articles on many of the points above.

Software and code

Policy information about availability of computer code

Data collection	No software was used.
Data analysis	Software developed for the proposed method BEAPR is available at Github: https://github.com/gxiaolab/BEAPR/wiki

For manuscripts utilizing custom algorithms or software that are central to the research but not yet described in published literature, software must be made available to editors/reviewers. We strongly encourage code deposition in a community repository (e.g. GitHub). See the Nature Research guidelines for submitting code & software for further information.

Data

Policy information about availability of data

All manuscripts must include a data availability statement. This statement should provide the following information, where applicable:

- Accession codes, unique identifiers, or web links for publicly available datasets
- A list of figures that have associated raw data
- A description of any restrictions on data availability

All data sets used in this study can be obtained from the ENCODE project website at http://www.encodeproject.org.

Field-specific reporting

Please select the one below that is the best fit for your research. If you are not sure, read the appropriate sections before making your selection.

☒ Life sciences ☐ Behavioural & social sciences ☐ Ecological, evolutionary & environmental sciences

For a reference copy of the document with all sections, see nature.com/documents/nr-reporting-summary-flat.pdf

Life sciences study design

All studies must disclose on these points even when the disclosure is negative.

Sample size	We obtained RNA-Seq and eCLIP-Seq data from the ENCODE consortium. As a standard practice of ENCODE for this project, two biological replicates were generated for each experiment.
Data exclusions	No data was excluded.
Replication	Experimental validations were carried out to verify bioinformatic findings.
Randomization	not applicable
Blinding	not applicable

Reporting for specific materials, systems and methods

We require information from authors about some types of materials, experimental systems and methods used in many studies. Here, indicate whether each material, system or method listed is relevant to your study. If you are not sure if a list item applies to your research, read the appropriate section before selecting a response.

Materials & experimental systems

n/a	Involved in the study
☐	☒ Antibodies
☐	☒ Eukaryotic cell lines
☒	☐ Palaeontology
☒	☐ Animals and other organisms
☒	☐ Human research participants
☒	☐ Clinical data

Methods

n/a	Involved in the study
☒	☐ ChIP-seq
☒	☐ Flow cytometry
☒	☐ MRI-based neuroimaging

Antibodies

Antibodies used	The following antibodies were used: anti-HIS antibody (Santa Cruz Biotech, sc-8036, 1:500 dilution), GEMIN5 antibody (Bethyl Lab, A301-325A, Lot# A301-325A-1, 1:200 dilution), β-Actin antibody (Santa Cruz, sc-47778, Lot # J2915, 1:500 dilution).
Validation	Manufacturer verification images available on their websites. Many publications have used these antibodies.

Eukaryotic cell lines

Policy information about cell lines

Cell line source(s)	HeLa cells from ATCC were used for reporter assays
Authentication	The cell line was aliquots of cells that were originally purchased from ATCC.
Mycoplasma contamination	The cell line tested negative for mycoplasma using Venor GeM Mycoplasma Detection Kit (Sigma-Aldrich, MP0025)
Commonly misidentified lines (See ICLAC register)	No commonly misidentified cell lines were used.
