# Supplementary material for: Allele-specific binding of RNA-binding proteins reveals functional genetic variants in the RNA
Source: Nat Commun. 2019 Mar 22;10:1338. doi: 10.1038/s41467-019-09292-w (PMC6430814; doi:10.1038/s41467-019-09292-w)
Supplement: Supplementary file 3 — Description of Additional Supplementary Files [file 41467_2019_9292_MOESM3_ESM.pdf]

Description of Additional Supplementary Files

File Name: Supplementary Data 1
Description: Primer sequences used in this study

File Name: Supplementary Data 2
Description: ASB events identified in HepG2 and K562 cells.
